# Black is the new orange: inline synthesis of silica-coated iron oxide nanoparticles produced *via* gas-phase in a matrix burner†

**DOI:** 10.1039/d5ra00808e

**Published:** 2025-05-08

**Authors:** Claudia-Francisca López-Cámara, Sabrina Schleich, Juliana Davoglio Estradioto, Paolo Fortugno, Mohammed-Ali Sheikh, Joachim Landers, Soma Salamon, Heiko Wende, Hartmut Wiggers

**Affiliations:** a Institute for Energy and Materials Processes – Reactive Fluids, University of Duisburg-Essen 47057 Duisburg Germany; b Center for Nanointegration Duisburg-Essen (CENIDE), University of Duisburg-Essen 47057 Duisburg Germany; c Mechanical Engineering Department, Eindhoven University of Technology Eindhoven 5612 AJ The Netherlands c.f.lopez.camara@tue.nl; d Eindhoven Institute for Renewable Energy Systems (EIRES), Eindhoven University of Technology Eindhoven 5600 MB The Netherlands; e Materials Science and Engineering Department, Northwestern University Evanston 60208 IL USA; f Faculty of Physics, University of Duisburg-Essen Duisburg 47057 Germany

## Abstract

Superparamagnetic iron oxide nanoparticles (IONPs) have a large range of applications, such as pollutant removal and inductive heating. Some of these applications benefit from coating the IONPs with silica (SiO_2_) to conserve their properties and/or prevent their aggregation; yet, the habitual synthesis methodologies require several steps, which limit their industrial scalability. In this work, we explore the capability to synthesize and stabilize oxidation-sensitive phases of IONPs *via* gas-phase flame synthesis as an alternative methodology that enables continuous operation. The addition of an inline quench gas nozzle—to avoid aggregation/agglomeration—and a coating nozzle is investigated to clarify their roles in contributing to the properties of the resultant coated IONPs. Three different quench and coating configuration heights above burner (HAB) are studied. The resultant synthesized Fe_*x*_O_*y*_|SiO_2_ core–shell nanoparticles are characterized using (scanning) transmission electron microscopy ((S)TEM), X-ray diffraction (XRD), Fourier-transform infrared spectroscopy (FTIR), elemental analysis, dynamic light scattering (DLS), Mössbauer spectroscopy, magnetometry, and energy-dispersive X-ray spectroscopy (EDX) from scanning electron microscopy (SEM). Results show that the synthesized nanoparticles presented a mixture of oxidation states—mainly magnetite (Fe_3_O_4_) and maghemite (γ-Fe_2_O_3_) phases—and a narrow primary particle size distribution. Quenching the IONPs early decreased the nanoparticle agglomeration/aggregation up to one order of magnitude. Moreover, homogeneous coating was achieved in all cases. Increasing the coating thickness helped reduce oxygen diffusion to the iron oxide core of the coated IONPs, conserving more magnetite phase in the coated IONP cores. These insights allowed us to conclude that targeted coated IONPs can be successfully produced through gas-phase synthesis using a flame reactor. In the near future, the long-term stability of IONP properties will be explored using this inline coating.

## Abstract 2

Flame synthesis process optimized to obtain (superpara)magnetic coated and uncoated maghemite and magnetite.

## Graphical abstract

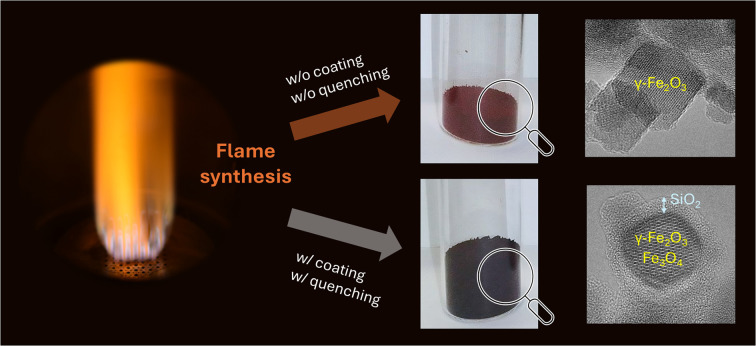

## 1. Introduction

Iron oxide nanoparticles (IONPs) have a large range of physico-chemical properties that make them highly interesting for many applications, such as food coloring (ranging from orange to black),^1^ pollutant removal,^2^ catalysis,^3^ and cancer therapy,^4,5^ ─ the latter utilizing the superparamagnetic properties of IONPs. Due to their large surface-to-volume ratio, nanoscale redox-sensitive materials like iron oxides are particularly susceptible to changes in the chemical environment. This can be useful for *e.g.*, removing pollutants, or disadvantageous if specific magnetic or catalytic properties of the IONPs are in the foreground, since they strongly depend on phase composition and the metal oxidation state.

IONPs are mainly synthesized *via* wet-chemical and gas-phase processes. For the latter, flame synthesis is a commonly employed and established process.^6–8^ Moreover, gas-phase flame synthesis methods for the production of specific nanoparticles can operate continuously, are highly reproducible and scalable,^3,9^ produce less waste, and lower the costs compared with other methods such as co-precipitation.^10^ Depending on the type of flame used and the respective experimental parameters, the reactants and formed particles can experience different equivalence ratios and temperature/time profiles influencing the resultant nanoparticle characteristics by *e.g.*, affecting their size, stoichiometry, phase composition, magnetic properties, and agglomeration. For example, the equivalence ratio and the temperature/time profile are crucial factors during IONP formation that enable the production of materials with a range of stoichiometries.^6,11–13^ Furthermore, thermal quenching of the gas-laden reactive flows containing synthesized nanoparticles is often employed to alter some of the aforementioned characteristics. When such quenching is applied early to flames, it is able to affect the combustion reactions. Depending on the desired applications, coating of the nanoparticles may be required. For the flame synthesis method, coating IONPs with silica (SiO_2_) is the most common method to stabilize IONPs by preventing changes in oxidation state and avoiding interparticle magnetic interaction while showing good biocompatibility and stability.^14^ Hence, combining inline quenching and coating of IONPs in the early stages of flame synthesis (*i.e.*, right after their flame synthesis) is a suitable approach for tuning their properties, protecting them from further changes in the oxidation state and thus, stabilizing their composition and magnetic characteristics.

A comprehensive summary of previous literature is shown in Table 1, portraying the publications on coated iron oxides *via* flame synthesis, as well as the precursors used, nanoparticle sizes, and magnetic properties information obtained from these studies. To expand on the findings from this literature, the present work explores the capability to synthesize and stabilize specific compositions of IONPs with respect to their magnetic properties *via* synthesis in a diffusion burner and the application of a downstream quenching and coating process. Quenching and coating effects are studied as (i) it is known that metal oxides release oxygen when heated, which means that particles being at higher temperature are oxygen deficient, and (ii) it is expected that a fast coating will impede the oxygen present in the surroundings to penetrate and further oxidize the iron as it is transported downstream. These effects are investigated both separately and together, with the aim to provide more insights on potential optimal synthesis parameters for superparamagnetic IONPs using gas phase flame synthesis. Furthermore, the magnetic behavior of the obtained materials is compared and reported, as it is a crucial factor for future applications.

**Table 1:** Comprehensive summary of literature using flame synthesis for silica-coated iron-oxide production. “prec.” stands for precursor; iron(iii) acetylacetonate (Fe(acac)_3_); iron pentacarbonyl (Fe(CO)_5_); tetraethyl silicate (TEOS); hexamethyldisiloxane (HMDSO); tetramethylsilane (TMS)

Ref.	Synthesis of	Core prec.	Shell prec.	Core size/nm	Shell thickness/nm	Magnetism
19	SiO_2_-coated γ-Fe_2_O_3_	Fe(acac)_3_ in xylene and acetonitrile	TEOS	4–21	3.5–5.5	20.8–73.6 emu g^−1^ (γ-Fe_2_O_3_) and 38.2–40 emu g^−1^ (SiO_2_-coated γ-Fe_2_O_3_). Magnetization recorded at 5 K
20	SiO_2_-coated γ-Fe_2_O_3_	Fe(CO)_5_	HMDSO	<10	<100	Mostly γ-Fe_2_O_3_, although they cannot rule out having produced magnetite (Fe_3_O_4_). Superparamagnetism over a wide temperature range
21	SiO_2_-coated γ-Fe_2_O_3_	Fe(CO)_5_	TMS	3–7	9–13	Superparamagnetic at room temperature. Blocking temperature at 40 K. ∼20 emu g^−1^. Magnetization recorded at 5 K
22 and 23	SiO_2_-coated Ag/γ-Fe_2_O_3_	Fe(acac)_3_ and silver acetate dissolved in 2-ethylhexanoic acid and acetonitrile	HMDSO	12–20.4	2–3	39.4–46.1 emu g^−1^ for Ag-contents *x* = 10–35 wt%, similar to pure γ-Fe_2_O_3_ core (38.4 emu g^−1^)
5 and 24	SiO_2_-coated γ-Fe_2_O_3_	Fe(acac)_3_ in dissolved in xylene and acetonitrile	HMDSO	19–22.4	2	Suggested threshold of >12 wt% SiO_2_ for hermetic or continuous coating. 32 emu g^−1^ (at 8 kOe) for SiO_2_-coated γ-Fe_2_O_3_ and coercivity of 0.1 kOe

## 2. Experimental setup

Iron pentacarbonyl, Fe(CO)_5_, is used as the precursor for the IONPs produced in this work as it has a high vapor pressure and fast decomposition kinetics in the flame. This means that after nucleation, IONPs undergo changing conditions depending on temperature and gas-phase composition towards the colder parts of the flame.^15^ Thus, this work uses these characteristics as an advantage to study the different resultant IONP phases. For the coating, silica (SiO_2_) was used for the reasons anteriorly described and tetraethyl orthosilicate (TEOS) was chosen as a precursor based on our previous studies.^16^

**Fig. 1:**
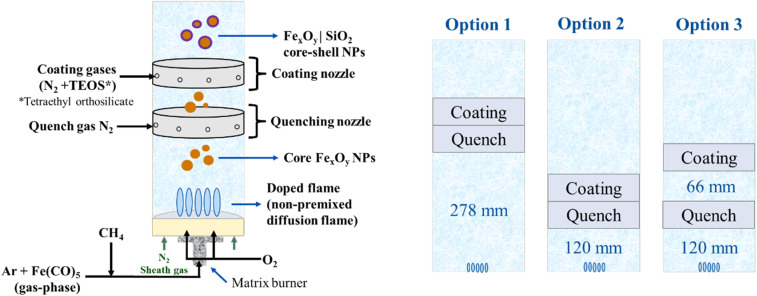
Reactor setup (left) and different nozzle configurations with spacings between the burner-nozzles marked (right).

The gas-phase reactor that was used accounts for a 1D matrix burner, capable of operating under premixed and diffusion flame conditions.^17^ In this work, only the diffusion flame mode was explored, where the inlet gas-phase Fe(CO)_5_ was injected together with the fuel (methane, CH_4_) and argon (Ar) as a dilutant. The oxidizer (oxygen, O_2_) is injected through separated channels on the burner. Once the reactant reaches the flame, it first decomposes into iron atoms followed by the formation of Fe_*x*_O_*y*_H_*z*_ molecular species, which nucleate, condense, and grow to form the resultant nanoparticles. An inline quench nozzle was added downstream to influence nanoparticle growth and agglomeration. Also, an inline coating nozzle was placed after the quenching (see Fig. 1) to investigate how coating of the IONPs helps protect early-formed IONPs. For the SiO_2_ coating, TEOS is vaporized and sent into a coaxial cylindrical coating nozzle downstream of the particle formation zone. Both inline quenching and coating nozzles have the same structural features and have a height of 30 mm each. These nozzles are replicas of the coating nozzle used in previous works,^16^ where the mass flow was adjusted to conditions that enabled intimate mixing with the particle-laden exhaust gas from the burner, as confirmed by fluid dynamic calculations.^18^ The burner/nozzles spacings are chosen based on the ones used in,^16^ as well as the expectancy for these cases to produce significantly different results. Moreover, the choice of placing the quench nozzle before the coating nozzle is to avoid TEOS decomposing too fast,^18^ which would lead to unwanted higher homogeneous nucleation of silica. Furthermore, “turning off” the coating nozzle in comparison to no built-in coating nozzle is not expected to make any difference on the resultant nanoparticles.

### 2.1 Synthesis and inline coating setup details

Fig. 1 shows a scheme of the experimental setup for the synthesis, quenching, and coating of IONPs with SiO_2_. The matrix burner is enclosed in a quartz tube (83 mm inner diameter) with the burner-nozzle configurations shown in Fig. 1. The burner consists of an array of non-premixed methane–oxygen diffusion flames (2 slm for CH_4_ and 4 slm for O_2_) and it is operated at 450 mbar abs. The Fe(CO)_5_ is transported in the gas phase by an Ar stream at 0.1 slm. This Fe(CO)_5_-carrying Ar stream is mixed with the CH_4_ stream and another Ar dilution stream of 13.5 slm, injecting a total of 17.6 slm through the designated fuel channels of the matrix burner. Moreover, N_2_ sheath gas is injected at 20 slm from the side flanges below the burner to cool the burner down and to help avoid water condensation in the reactor (not pictured in Fig. 1). For the same purpose, the burner and the top of the reactor chamber are heated with water jackets at 40 °C and 70 °C, respectively.

For the inline quench nozzle, 10 slm N_2_ were used in all the experiments except for the control experiment (Table 2, Case 0), which did not have any flow through the coating nor quenching nozzles. For the coating, tetraethyl orthosilicate (TEOS) is used as a reactant, which is introduced into the system using a vapor delivery module (VDM). The VDM provides the opportunity for controlled evaporation and mixing of the liquid coating precursor. For a consistent comparison between the different configurations and cases, the coating precursor mass flow rate was set to 1 g h^−1^ for the coating cases except for Case 2CC, where we intended to determine if increasing the flowrate of the coating precursor to 2.5 g h^−1^ would lead to thickening of the shell compared with Case 2C.

**Table 2:** Study cases. For Case 2, since no coating is applied, the same result is expected for configurations 2 and 3. The coating precursor (TEOS) was always set at 1 g h^−1^ except for Case 2CC, which was 2.5 g h^−1^ “Opt.” stands for “Option” (based on Fig. 1). The numbers in the case name correspond to the configuration used and the “C” corresponds to the coating applied. The two values on the PSD TEM correspond to the count median diameter from the lognormal distribution (logN) and the cumulative fraction = 0.5 (cumul.), respectively

Case	0	1	2	1C	2C	3C	2CC
Configuration	—	Opt. 1	Opt. 2/3	Opt. 1	Opt. 2	Opt. 3	Opt. 2
Quenching nozzle	OFF	ON	ON	ON	ON	ON	ON
Coating nozzle	OFF	OFF	OFF	ON	ON	ON	ON (×2.5)
Theoretical coating thickness/nm	—	—	—	2.3	2.2	2.2	4.2
**Resultant BET specific surface area**							
BET SSA/(m^2^ g^−1^)	91.5	68.7	84.3	64.2	89.0	80.2	68.1
**Resultant IONP particle size (TEM) and agglomerate/aggregate size (DLS)**							
PSD TEM/nm (logN)	14.4	18.2	11.3	20.0	13.3	16.7	20.6
PSD TEM/nm (cumul.)	12.1	15.5	9.84	16.9	10.9	14.4	17.6
PSD DLS/nm	1310	76	43 & 105	1116	85	32 & 78	167
**Resultant elemental analysis (EDX from TEM | SEM) for coated cases**							
Oxygen/at%	73.3|67.33	66.3|65.46	73.8|65.98	69.16|66.72	71.6|69.55	51.8|68.42	70.1|69.49
Silicon/at%	—	—	—	0.98|0.85	6.2|5.44	3.4|5.28	12.6|12.24
Iron/at%	26.7|32.67	33.7|34.54	26.2|34.02	29.84|32.43	22.3|25.00	19.9|26.30	17.4|18.28
**Resultant IONP phases (Mössbauer spectroscopy)**							
Maghemite/%	99 ± 4	97 ± 3	99 ± 3	95 ± 3	80 ± 3	77 ± 3	58 ± 3
Magnetite/%	1 ± 4	3 ± 3	1 ± 3	5 ± 3	20 ± 3	23 ± 3	42 ± 3

To estimate the expected coating thickness from the given flowrates, the first experiments were performed without coating precursor (Cases 1 and 2). These study cases represent two different conditions of distances between the burner and the quenching nozzle. The theoretically expected shell thicknesses for the coated study cases are shown in Table 2 (Cases 1C, 2C, 3C and 2CC) based on the obtained particle sizes, the specific surface area, and assuming full conversion towards particles. The TEOS coating flow rate (1 g h^−1^ for all cases and 2.5 g h^−1^ for Case 2CC) was mixed with N_2_ dilution gas to provide a total coating flow of 5 slm to the system.

Finally, quench nitrogen (N_2_) is injected at 70 slm before the filter to cool down the reactor gases (not pictured in Fig. 1).

### 2.2 Particle characterization

Both uncoated and coated IONPs were characterized to investigate their morphology and properties. For all cases, their analyses were carried out within three weeks of nanoparticle synthesis. For conventional transmission electron microscopy (TEM) as well as scanning TEM (STEM) analysis, a JEOL JEM-2200FS microscope was used. Sample preparation involved thoroughly dispersing the nanoparticles in ethanol by 20′ of sonification, which were deposited on a lacey carbon support on a copper TEM grid afterwards. Fiji-ImageJ software^25^ was used for the particle size distribution and particle counting analyses. The presence of iron (Fe), oxygen (O), and silicon (Si) at selected areas and particles in STEM images was determined by energy-dispersive X-ray (EDX) analysis (Oxford EDX detector) using both TEM and scanning electron microscopy (SEM, Zeiss LEO 1530). For the latter, powder was directly smeared on a carbon adhesive tape that was then put on a SEM sample support in order to proceed with EDX. Moreover, SEM images of the powder's surface morphology were also taken. For that, sample preparation consisted of dissolving a spatula tip of the nanoparticle powder in 5 mL of ethanol and ultrasonicating the mixture for 5 min. Then, it was drop-casted on a silicon substrate, allowing each drop to dry before applying the next one. A total of three drops were drop-casted for each sample. X-ray diffraction (XRD) patterns were obtained with a Rigaku Smartlab X-ray diffractometer using Cu K_α_ radiation. The Fourier-transform infrared spectroscopy (FTIR) spectra were measured within wavenumbers ranging from 4000 to 400 cm^−1^ using attenuated total reflection mode with an FTIR spectrophotometer (Bruker Vertex 80). The Brunauer–Emmett–Teller (BET) specific surface area analysis (SSA) was done using nitrogen adsorption (Anton Paar Nova 800) after degassing the samples for 960 min at 120 °C under vacuum. Furthermore, the materials were analyzed by elemental analysis (ELTRA® ELEMENTRAC CS-i) and dynamic light scattering (DLS; Zetasizer Nano ZS with a 633 nm laser). DLS was used to quickly, easily, and reliably size the aggregates.

To analyze the quality of the obtained coatings, the leaching of iron out of the powdered samples in ultrapure hydrochloric acid (HCl) solutions was quantified *via* atomic absorption spectroscopy (iCE 3500 Series, Thermo Fisher Scientific). Ultrapure 34% HCl (Carl Roth) was used and diluted with ultrapure water to 4 M. Then, 2 mL of the diluted acid were added to 5 mg powder of each sample and kept in a water bath at 30 °C for 1.5 h. Afterwards, the resulting dispersions/solutions were vacuum filtrated with a silica filter (Whatman GF 10). The filter and glass containers were washed out with additional ultrapure water (in total, 16 mL), yielding a total of ∼18 mL of sample solution.

To characterize the particles' magnetic structure and composition, Mössbauer spectra were recorded at 5 K in an applied field of 8 T along the γ-ray propagation direction using a magnet cryostat (Spectromag 4000-10, Oxford Instruments). Additional information on the particles' relaxation dynamics were extracted from spectra recorded between 5–300 K in a closed-cycle cryostat (SHI-850-5, Lake Shore Cryotronics). All measurements were performed in transmission geometry and constant acceleration mode on ≈20–30 mg cm^−2^ of nanoparticle powder mixed with chemically inert boron nitride and pressed into cylindrical discs. Magnetometry measurements were performed with the vibrating sample magnetometer (VSM) option for the Quantum Design PPMS DynaCool. Field-dependent *M*(*H*) magnetization curves were recorded up to fields of ±9 T at temperatures of 5 K and 300 K, while temperature-dependent measurements were performed following the zero-field-cooled/field-cooled (ZFC–FC) protocol, with an applied magnetic field of 10 mT.

## 3. Results and discussion

The analysis and discussion of the results is presented in two parts. First, the discussion focuses on the effect of quenching and coating and how that affects the general aspects of the nanoparticles (*i.e.*, size, morphology, surface area, agglomeration). Then, the discussion shifts toward the magnetic behavior of the resultant IONPs and how this is related to the applied quenching and coating using the different nozzle configurations from Table 2.

Before moving forward, it is crucial to define that agglomeration and aggregation are two different processes and as such, this work uses the related different wording accordingly. As clarification, during aggregation, sinter necks form between individual particles, which can be influenced by changing the temperature field (*e.g.*, by quenching). During agglomeration, point contacts are formed due to van der Waals interactions. These cannot be prevented but can easily be loosened again during further processing.

### 3.1 Effects on general IONP characteristics

**Fig. 2:**
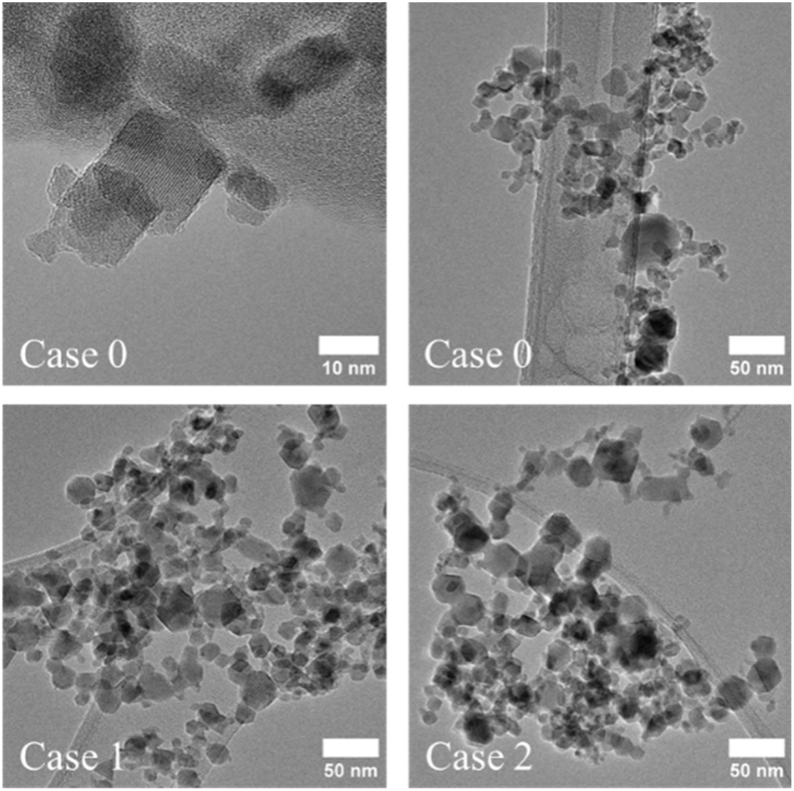
TEM images of the coated IONPs (Cases 0, 1, and 2).

**Fig. 3:**
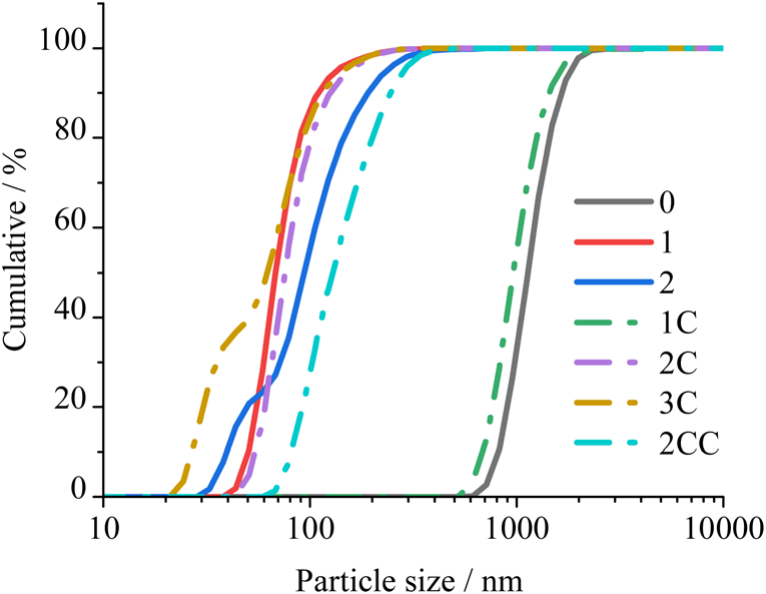
Cumulative particle size from DLS. Full lines refer to the experiments without coating, while dashed-dotted ones are for the experiments generating coated nanoparticles. Refer to online publication for colors.

Looking into the effect of quenching at different heights when no coating is used (Cases 1 and 2, Fig. 2), the cumulative DLS from Table 2 and Fig. 3 confirm the expectation that aggregation/agglomeration is reduced as quenching occurs closer to the burner (*i.e.*, smoother slope of the graph in Fig. 3, Case 2). This is because quenching closer to the region where IONPs are made allows less time for them to grow and/or agglomerate. Hence, it is not surprising that the largest agglomerates are produced (≈1 μm) when no quenching nor coating is used (Case 0). Comparing Case 0 against Cases 1 and 2, quenching the particles further from the burner leads to bigger particles compared with unquenched particles, while a slightly opposite effect occurs when quenching them closer to the burner. However, the agglomerates/aggregates from Case 0 are one order of magnitude bigger than the ones for Cases 1 and 2. This means that even though the quench gas does not significantly change the primary particle diameter, it does play a major role for the aggregate/agglomerate size, which is also observed for the coated cases.

**Fig. 4:**
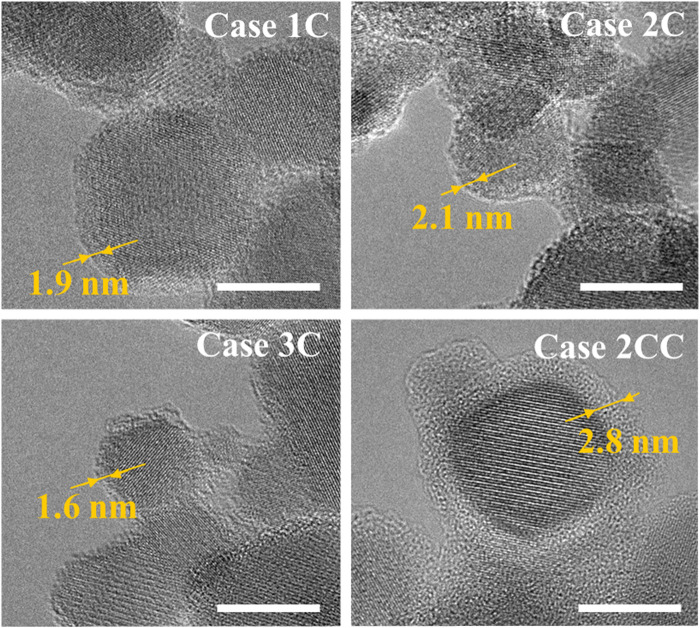
TEM images of the coated IONPs. The scale bar (white line) represents 10 nm. The thickness values correspond to the specific images and not the median for the entire sample.

For the coating cases (Cases 1C, 2C, 3C, and 2CC), homogeneous amorphous SiO_2_ coating of the IONPs has been achieved in all cases (Fig. 4), whose composition has been confirmed by EDX, EDX line scans, and FTIR spectra (ESI, Sections S.4 and S.6†). Overall sample measurements confirming the coating existence have also been performed *via* atomic absorption spectroscopy (see Section 3.2 Coating quality testing). Looking into the coated IONPs' cases, the change in particle and agglomerate/aggregate sizes is not significant in any of the cases except for Case 1C. This could be because of the lower temperature at the quenching and coating nozzle, as they were placed at the furthest position from the flame compared with the other cases. This increases the TEOS decomposition time, which might lead to the effect that the smaller aggregates are “glued” together (Case 1). Looking into the overall BET SSA results (Table 2), the coated cases display a decrease in SSA compared with their counterparts, as expected due to an increase in particle size when the coating is present. This trend is also followed when more coating is present (Case 2C *vs.* Case 2CC). Notice that the theoretical SiO_2_ coating thickness for Case 2CC is 4.2 nm, but the experimental measurements show a median of ≈6.3 nm for Case 2CC (ESI, Fig. S2†). For the other coated cases (1C, 2C and 3C), the coating was too thin to be measured *via* TEM images. The images in Fig. 4 show examples of homogeneous coatings, and the thickness values correspond to the specific images, not the average for the entire samples.

Related to the temperature profile, the gas-flow temperature after the quench nozzle is estimated to be ≈300–350 °C. The glow of the particle-laden flame and the area directly above it (and before the quench nozzle) is really bright, yet it is unclear if it originates from the particles themselves. If it originates from the particles by thermal radiation, these would be at ≈1000 K. The main expected size-dependent effect is the aggregation of nanoparticles, as the effect on agglomeration is negligible.^26^

Generally, the particle size (distribution) obtained from TEM (Table 2 and ESI, Section S.2†) is moderately affected by the quenching and coating of the nanoparticles. Yet, the agglomerate/aggregate sizes (Fig. 3) are seen to be heavily affected by quenching, showing that quenching does substantially help to reduce the agglomeration/aggregation of IONPs.

Regarding the composition of the IONPs, a dark brown/black powder was obtained for all cases, with the coated samples generally looking darker than the uncoated ones, and especially for Case 2CC. This was already an initial suggestion that the composition of all samples could be a mixture of iron phases that included magnetite—which is black—, and that the coated samples probably contained more magnetite than the uncoated ones as they were darker. After XRD and FTIR, it was confirmed that all the cases contain a mixture of mainly maghemite and magnetite (ESI, Sections S.5 and S.6†), and XRD qualitatively showed that Case 2CC contains more magnetite than the rest of cases due to the lack of peaks at 23.8 and 26.2 degrees (similar to previous literature^12^). Also, the XRD data confirm that the change in primary particle size between the different samples is insignificant. Due to the similarity of the FTIR and XRD results between the two phases, accurate quantitative data for them was obtained *via* Mössbauer spectroscopy (Table 2 and discussed later). Moreover, carbon analyses confirm the absence of hydrocarbon incorporations in both uncoated and coated cases (see carbon elemental analysis results in ESI, Section S.7†).

### 3.2 Coating quality testing

For calculating the relative amount of leached iron, several assumptions were made. First, the relative amount of silica in the powdered samples was estimated based on the TEM-EDX. Then, the absolute mass of iron in each sample was calculated based on the phase composition estimated from Mossbauer spectroscopy. Subsequently, the maximum possible amount of dissolved iron was compared to the measured values from atomic absorption spectroscopy (Table 3).

**Table 3:** Results from leaching the nanoparticles from all the studied cases

Case	Weighted powder/mg	Relative iron oxide content/wt%	Iron mass/mg	Max. iron concentration/mg L^−1^	Measured iron concentration/mg L^−1^	Relative amount leached/%
0	5.06	100	3.54	197.72	187.7	94.9
1	5.23	100	3.66	203.21	199	97.9
2	5.08	100	3.55	197.38	187.3	94.8
1C	5.25	99	3.64	202.29	196.2	96.9
2C	5.12	93	3.35	186.11	85.7	46
3C	5.12	95.5	3.45	191.66	77.4	40.4
2CC	5.24	84	3.12	173.33	8.1	4.7

The results show that all cases leach part of the iron oxide cores. For the uncoated particles (Cases 0, 1, and 2), almost all the iron is leached; however, coated particles generally show a reduction (except Case 1C). The leaching results of Cases 2C and 3C could mean that some iron oxide particles are coated and some are free, Janus-like, due to incomplete mixing of the core aerosol stream with the TEOS stream, which was quantitatively shown in previous literature for flame-made silica-coated metal oxides.^22,27,28^ For Case 2CC, the iron oxide cores show the least relative amount leached (4.7%), which supports the presence of an effective coating (using an overall/“bulk” sample measurement) that shields the iron oxide cores, as suggested by the repeated Mössbauer results.

### 3.3 Effects on IONPs magnetic properties

One of the main properties that showcases the application appeal of IONPs is their magnetic behavior, which was analyzed *via* Mössbauer spectroscopy as well as field- and temperature-dependent magnetometry.

**Fig. 5:**
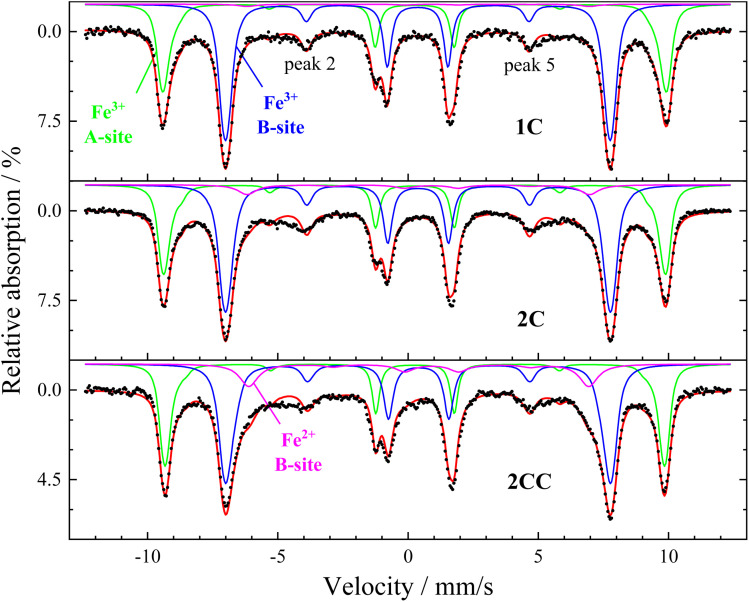
Mössbauer spectra recorded at 5 K in a magnetic field of 8 T applied parallel to γ-ray propagation direction, showing contributions of A-site (green), B-site Fe^3+^ (blue), and B-site Fe^2+^ (pink). Refer to online publication for colors.

**Fig. 6:**
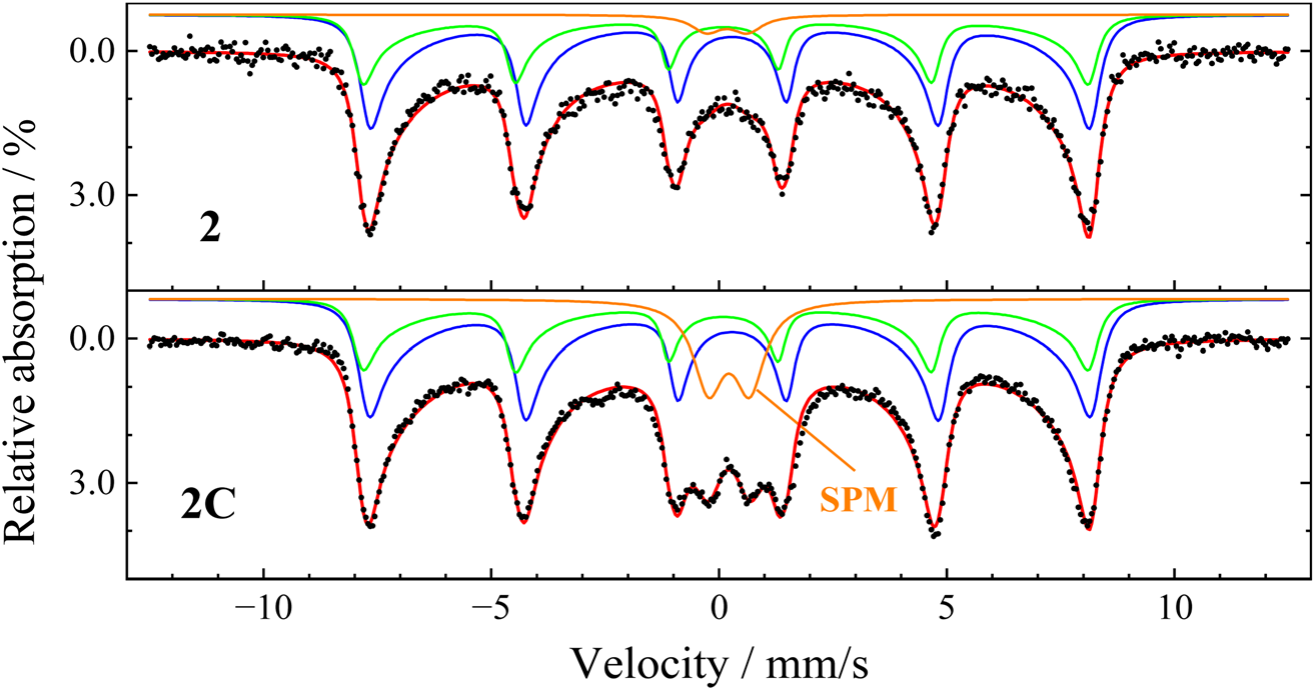
Mössbauer spectra recorded at 300 K, showing contributions of the iron ions on A- (green), B-sites (blue), and superparamagnetic nanoparticles (orange). Due to the stronger superposition and the deformation of the spectral structure by superparamagnetic relaxation, B-site contributions of Fe^2+^ and Fe^3+^ were not reproduced individually. Refer to online publication for colors.

To analyze the magnetic structure and composition for both uncoated and coated IONPs, Mössbauer spectra were recorded. The spectra display a typical structure for a mixture of the ferrimagnetic spinel iron oxides magnetite and maghemite in agreement with the XRD data (Fig. 5, recorded at 5 K in a magnetic field of 8 T). Here, two sextet subspectra represent Fe^3+^ ions in octahedral (B-site, blue) and tetrahedral (A-site, green) surroundings, with a minor third contribution of B-site Fe^2+^ (pink) of a lower hyperfine magnetic field and higher isomer shift.^29^ Sextet subspectra were reproduced *via* narrow hyperfine field distributions using the “Pi” program package.^30^ The magnetite–maghemite composition of the particles is reflected in the relative spectral area of the Fe^2+^ subspectrum: extracted maghemite and magnetite fractions are listed in Table 2. It is evident that bare particles (Cases 0, 1, and 2) contain (almost) no magnetite within the error bar. Case 1C does not show a relevant magnetite fraction either, while ≈20% of magnetite are found for Cases 2C and 3C, indicating that the magnetite phase can be conserved when quenching and coating IONPs closer to the burner. We attribute the increased proportion of magnetite to the expectation that a significant proportion of magnetite is present under the process conditions close to the burner, considering the temperature-pressure phase diagram for the iron/oxygen system.^31^ It should be highlighted that, since the maximum theoretical saturation magnetization values for magnetite and maghemite are 98 A m^2^ kg^−1^ and 82 A m^2^ kg^−1^, having more magnetite content is advantageous for achieving a higher magnetic moment of the superparamagnetic particles. Case 2CC, which has higher coating thickness as we doubled the TEOS concentration, exhibits >40% of magnetite, demonstrating the ability of our approach to conserve substoichiometric phase compositions during and after preparation, especially when considering the aging time of ≈2–3 weeks between particle synthesis and Mössbauer spectroscopy characterization. These findings align with previous literature on non-flame made silica-coated iron oxide nanoparticles,^29^ which show that silica coating not only conserves magnetite, but it is also known to tune particle interaction to different degrees depending on the shell thickness *e.g.*, comparing Cases 2 and 2C in the current study (Fig. 6). Moreover, in order to check for the aging on the nanoparticles, Cases 2C and 2CC were taken as examples and further Mössbauer spectroscopy measurements were performed of these samples about 9 months later, while keeping the samples under ambient conditions. These measurements indicated a decrease in magnetite concentration from 42 ± 3% to 30 ± 3% for Case 2CC, and 20 ± 3% to 16 ± 3% for Case 2C, showing only moderate further oxidation and no relevant change in magnetic structure or spin canting of the particles.

Additionally, we were able to discern more detailed information on the magnetic alignment of the particles. The relative spectral intensity of peaks 2 and 5 directly reflects the spin canting angle, referring to the average angle between magnetic field and spin direction, where zero intensity (or a canting angle of 0°) represents the state of magnetic saturation, with all magnetic moments being in colinear alignment to the magnetic field. While all studied cases display a relatively comparable and moderate degree in spin canting indicating weak interaction between the particles (ESI, Section S.8†), larger spin canting angles of *θ*_A_ ≈ 22° and *θ*_B_ ≈ 32° are observed for Case 0 for A- and B-site contributions, respectively. As the formation of larger agglomerate structures was observed for Case 0, this can likely be explained by direct surface contact and sintering of the primary particles, leading to stronger magnetic frustration at the particle surface. Previous literature also report on investigations towards avoiding this effect.^21^ Thus, this indirectly demonstrates that controlling the agglomeration behavior of the nanoparticles *via* quenching and “freezing” the respective oxidation state is a strategy to finetune the magnetic properties of the generated iron oxide nanoparticles.

To gather additional information on the particles' magnetic relaxation dynamics towards superparamagnetism, further spectra without an external magnetic field were measured between 5 and 300 K, with two exemplary spectra recorded at 300 K (Fig. 6). A growing deformation of the previously symmetric absorption lines is observed, which is caused by beginning Néel-type relaxation when the thermal energy is sufficiently high to enable slow relaxation of the nanoparticle magnetic moment between easy magnetic directions. Smaller particles of each sample with lower volume—and thereby lower magnetic anisotropy energy—will perform fast superparamagnetic relaxation (compared with the Mössbauer spectroscopy timescale of ≈5 ns), leading to the collapse of the sextet structure and the observation of a superparamagnetic doublet (SPM).^29,32^ Comparing Case 2 and Case 2C (Fig. 6), which are identical regarding synthesis parameters except for the coating, uncoated particles in Case 2 display a slightly lower asymmetric deformation and a far smaller doublet contribution of only ≈3% compared with 13% for the coated particles of Case 2C. This effect could be assigned to the absence of direct surface contact and the higher distance between coated particles in Case 2C, preventing direct surface exchange and decreasing magnetic dipolar interaction, which was reported to slow down superparamagnetic relaxation.^29,32^

A similar general trend is observed in temperature-dependent ZFC–FC magnetization measurements (Fig. S16,† indicating a partially superparamagnetic state of all samples up to 300 K). It is noteworthy that a characteristic feature in the magnetization curves at *ca.* 100 K assigned to the magnetite Verwey temperature is only found for Case 2CC, for which Mössbauer spectroscopy revealed the highest remaining magnetite fraction, further supporting this observation. It is well known that limited particle diameters and the beginning of the oxidation of magnetite nanoparticles can lead to a shift of the Verwey temperature, normally *ca.* 120 K, to lower temperatures and to a broadening of the transition.^33^

**Fig. 7:**
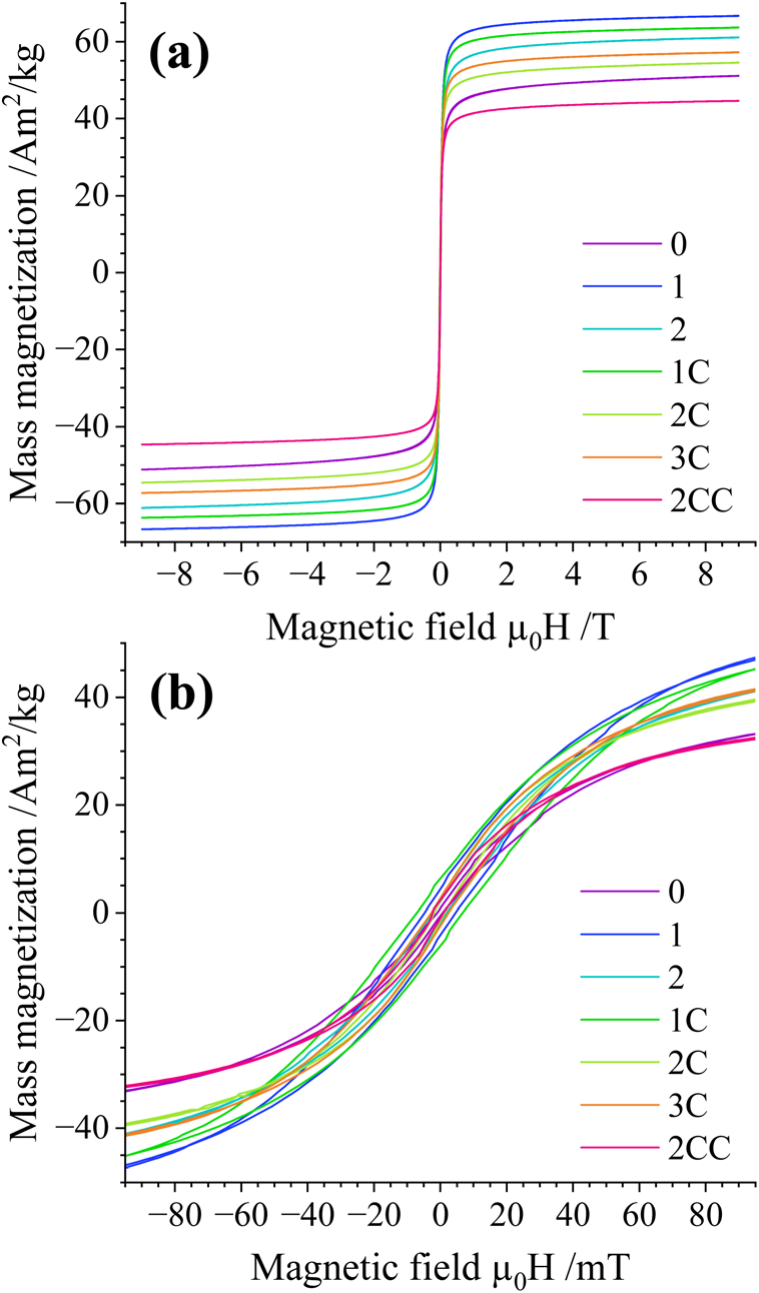
(a) *M*(*H*) magnetization curves recorded at 300 K and (b) zoom-in of (a). Magnetization values are normalized to the total sample mass. Refer to online publication for colors.

**Fig. 8:**
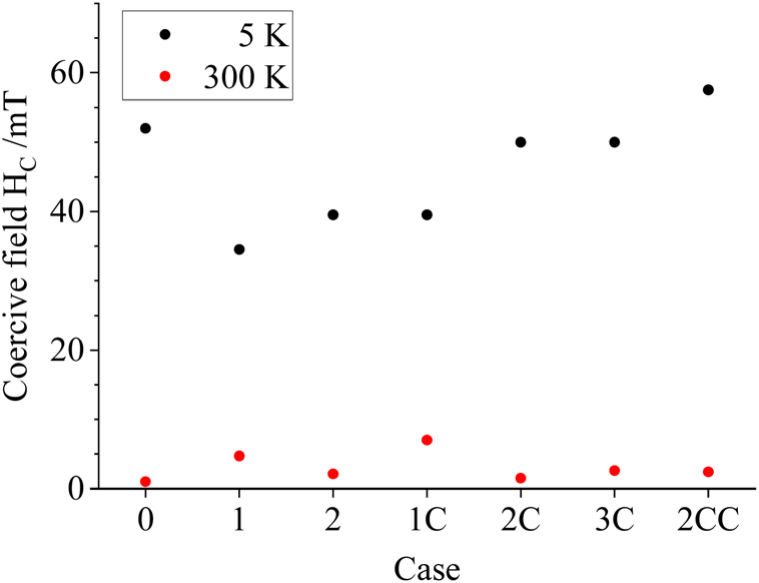
Coercive fields *H*_C_ extracted from the *M*(*H*) curves recorded at 5 K (black) and 300 K (red). Refer to online publication for colors.

General magnetic properties of the uncoated and coated particle powders were carried out *via* field-dependent magnetization measurements. The particles display comparable hysteresis behavior with low remanence and coercivity (*H*_C_), again verifying a partial superparamagnetic state at 300 K (Fig. 7 and 8). More details on the ZFC–FC magnetization measurements and field-dependent (*M*(*H*)) magnetization curves recorded at 5 K are shown in the ESI, Section S.8.† Based on these results, the magnetization values obtained from this work for non-coated particles at 5 K are in the upper end of the reported values from the literature;^19,22,23^ for coated particles, the magnetization values are only comparable with the literature ones when the coating is thick enough, as observed for Case 2CC (Table 1). Moreover, a gradually increasing trend in *H*_C_ is observed for all particles for Cases 1 to 2CC, which could originate from the higher conserved magnetite fraction in the coated samples in combination with slightly higher reported magnetic anisotropy values of magnetite compared with maghemite.^34^

**Fig. 9:**
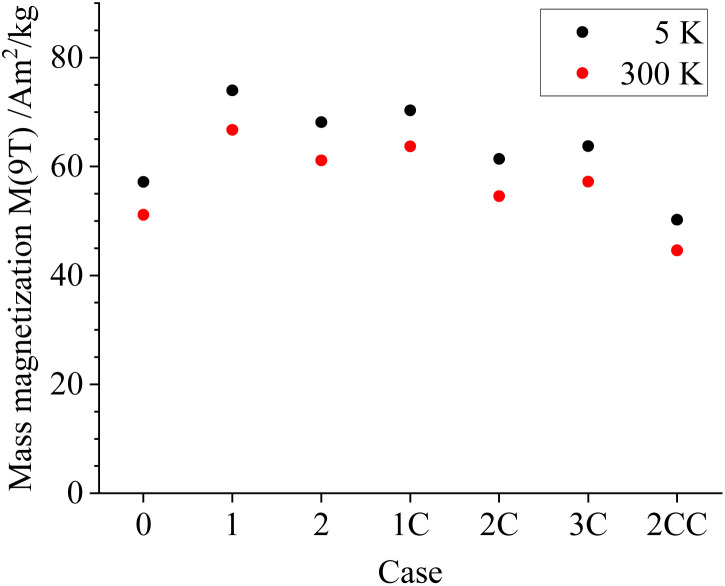
High-field magnetization values *M* (9 T) extracted from the *M*(*H*) curves recorded at 5 K (black) and 300 K (red). Magnetization values are normalized to the total sample mass. Refer to online publication for colors.

Fig. 9 shows the high-field magnetization values (*M* (9 T)) close to saturation, illustrating a slightly higher magnetization for larger particle magnetic cores prepared *via* Option 1 (*i.e.*, Cases 1 and 1C). This is likely connected to their lower specific surface compared with the remaining cases, which often displays frustrated magnetic structures, being difficult to align even in high magnetic fields. At the same time, a moderate decrease in *M* (9 T) takes place upon coating the particles with silica, reaching values of about 50 A m^2^ kg^−1^ for Case 2CC. Based on the limited decrease in magnetization, the silica density seems to be lower compared with bulk density values of about 2.2–2.6 g cm^−3^ when considering the silica volume fraction extracted from shell thicknesses from TEM analysis. This could be explained by having a porous silica nanostructure, as reported in similar nanoparticle silica coating works for non-flame made silica-coated iron-oxide nanoparticles^35^ and plasma-made silica-coated carbon structures.^36^

## 4. Conclusions

This work examines the parameters that benefit the stabilization and magnetic properties of iron oxide nanoparticles (IONPs) with silica (SiO_2_) coating, focusing on the role of quenching and nozzle positioning above the matrix-burner flame reactor. By introducing nitrogen quenching and a TEOS-based silica coating at different stages, the synthesized Fe_*x*_O_*y*_|SiO_2_ core–shell nanoparticles exhibit shell thicknesses between 1.5 nm and 6.3 nm, depending on the study case. Quenching significantly reduced particle aggregation by one order of magnitude, while early and thicker silica coatings preserved higher magnetite content by limiting the core's oxidation. Thus, the location/temperature for the coating is not arbitrary, and quenching the particles before coating them also plays a key role in the process. To our knowledge, this is the first study that produces mixtures of coated magnetite and coated maghemite nanoparticles, and demonstrates and quantifies the presence of these phases while studying the effect of quenching. Thus, we showed that it is possible to “freeze” the oxidation state of the nanoparticles at the site of SiO_2_ addition by means of the coating. Moreover, this work also shows that more coated magnetite phase can be obtained when the particles are coated with thicker shells.

Magnetic characterization revealed partially superparamagnetic behavior up to 300 K, with high-field magnetization values of about 50–65 A m^2^ kg^−1^, showing a moderate decrease due to the non-magnetic shell upon higher coating thickness. Uncoated IONPs (Case 0) exhibited larger spin canting angles, likely due to the direct surface contact and sinter bridge formation of the bare IONPs and the potential formation of sinter bridges due to the high agglomeration/aggregation of the bare IONPs. Thicker silica shells reduce magnetic frustration, with a minimum shell thickness of ∼6.3 nm necessary for effective silica-coating-shell iron-oxide nanoparticle. Future work will focus on more quantitative coating quality assessment, long-term stability, silica shell porosity, and scalability for potential applications.

## Data availability

The data supporting this article have been included as part of the ESI.†

## Conflicts of interest

The authors declare that they have no known competing financial interests or personal relationships that could have appeared to influence the work reported in this paper.

## Supplementary Material

RA-015-D5RA00808E-s001
