# Supplementary material for: Black is the new orange: inline synthesis of silica-coated iron oxide nanoparticles produced via gas-phase in a matrix burner
Source: RSC Adv. 2025 May 8;15(19):15121–30. doi: 10.1039/d5ra00808e (PMC12059999; doi:10.1039/d5ra00808e)
Supplement: RA-015-D5RA00808E-s001 [file RA-015-D5RA00808E-s001.pdf]

Supplementary Information (SI) for RSC Advances.
This journal is © The Royal Society of Chemistry 2025



# Supplemental Information

S.1. Iron oxide samples images

S.2. Particle Size Distribution (PSD) plots from TEM images

S.3. Agglomerate size distribution plots from DLS

S.4. Line profile from STEM images

S.5. XRD diffractograms

S.6. Fourier-transform infrared spectroscopy (FTIR) spectra

S.7. Elemental content analysis

S.8. Magnetic characterization

S.9. SEM/EDS characterization

## S.1. Iron oxide samples images

Figure S1 show three different iron oxide samples obtained from different cases using the flame-reactor employed in this work. The different colors shown are characteristic of the different oxidation states of iron, ranging from orange to brown and black.

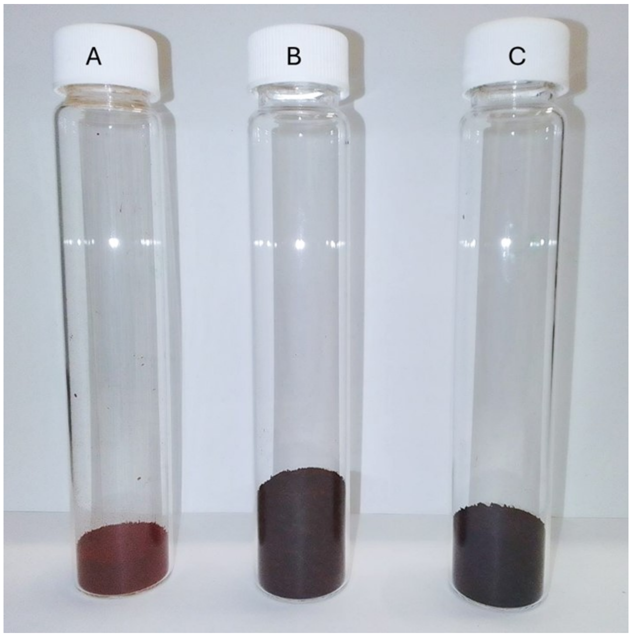


*Figure S1. Examples of iron oxide powders obtained via flame-synthesis. Color of the samples range from dark orange to brown/black due to their different oxidation state, as silica shell is too thin to change bulk powder color. From left to right (A to C): Case 0, 3C, and 2CC.*

## S.2. Particle Size Distribution (PSD) plots from TEM images

The eight subfigures in Figure S2 show the particle size distribution plots for each of the uncoated and coated synthesized nanoparticles with a log-normal distribution. These plots are based on 200 nanoparticles counting from the TEM images.

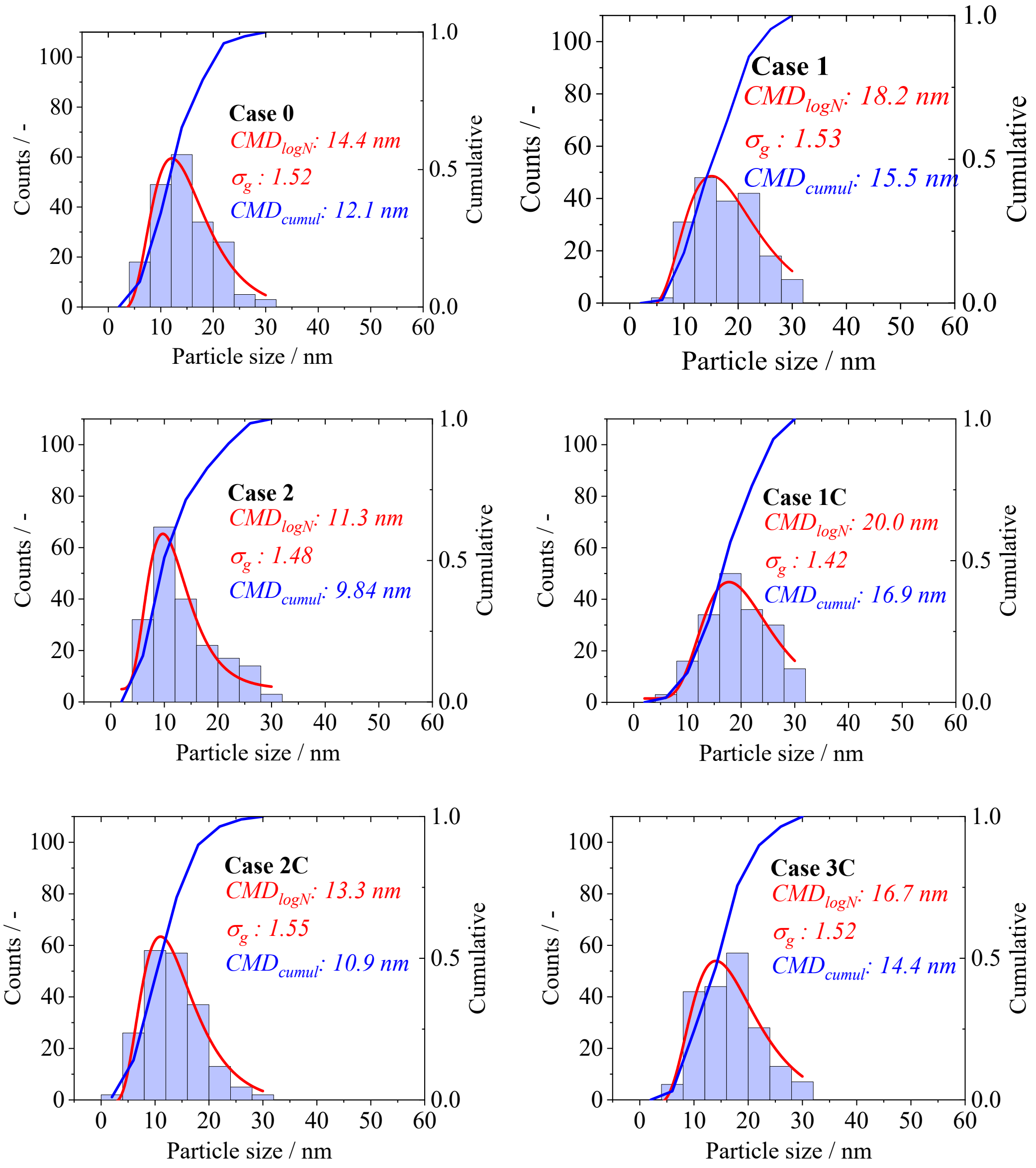


*Figure S2. Particle size distribution of synthesized nanoparticles based on particle count from TEM images. CMD stands for count median diameter (from the lognormal distribution and the cumulative fraction = 0.5, logN and cumul, respectively) and $\sigma_g$ is the geometric standard deviation.*

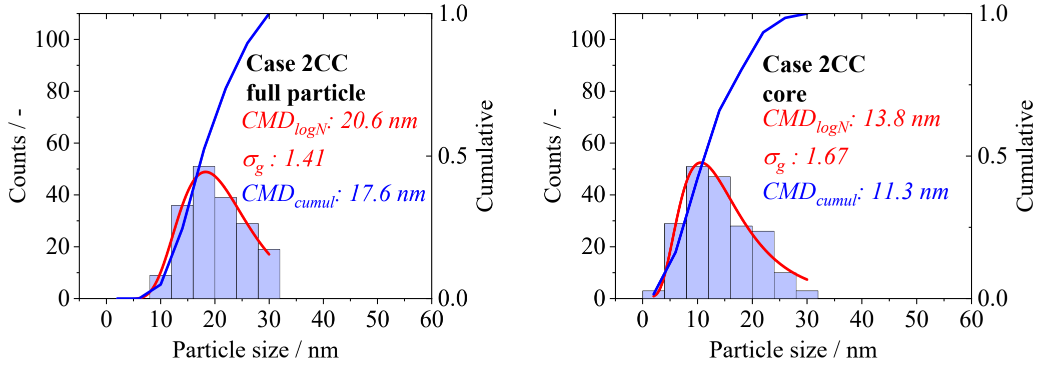


*Figure S2 (continuation). Particle size distribution of synthesized nanoparticles based on particle count from TEM images. CMD stands for count median diameter (from the lognormal distribution and the cumulative fraction = 0.5, logN and cumul, respectively) and $\sigma_g$ is the geometric standard deviation.*

## S.3. Agglomerate size distribution plots from DLS

The seven subfigures in Figure S3 show the agglomerate size distribution plots for each of the synthesized nanoparticles. Three measurements were performed for each of the synthesized nanoparticles and the average of them are the results displayed. The DLS is limited for polydisperse nanoparticles although the lognormal fit overcomes this difficulty by not considering the biggest agglomerates. Cases 2 and 3C required two lognormal fitting curves.

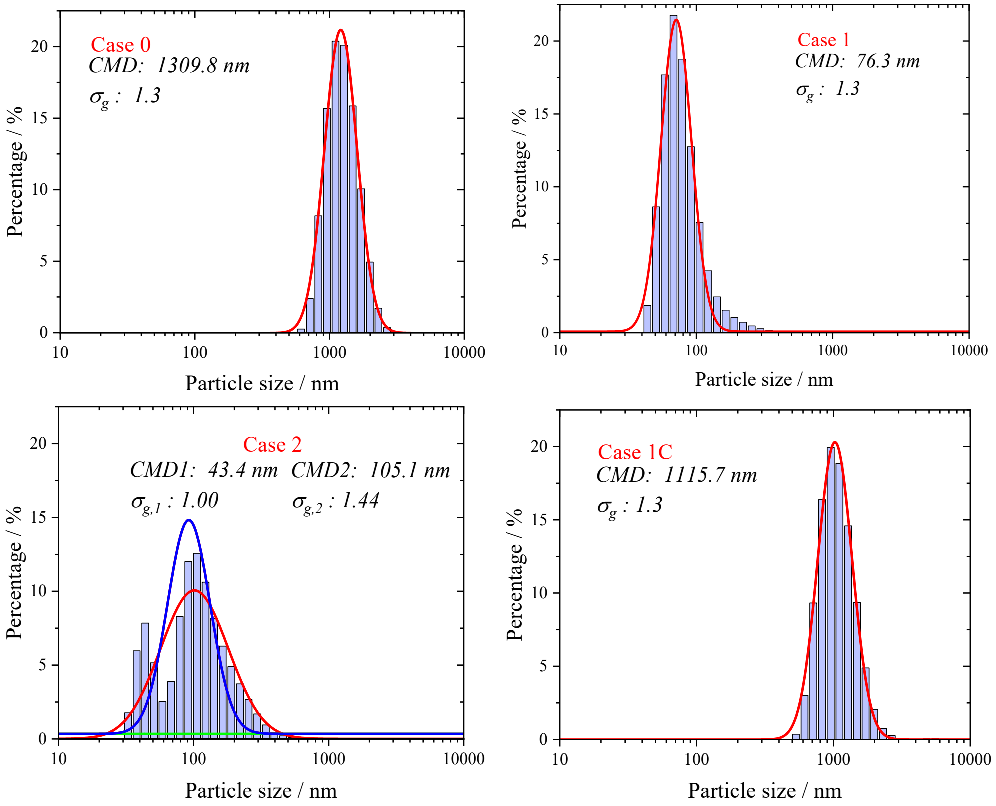


*Figure S3. Particle size distribution from DLS. Lognormal deviations and median diameters shown for each case.*

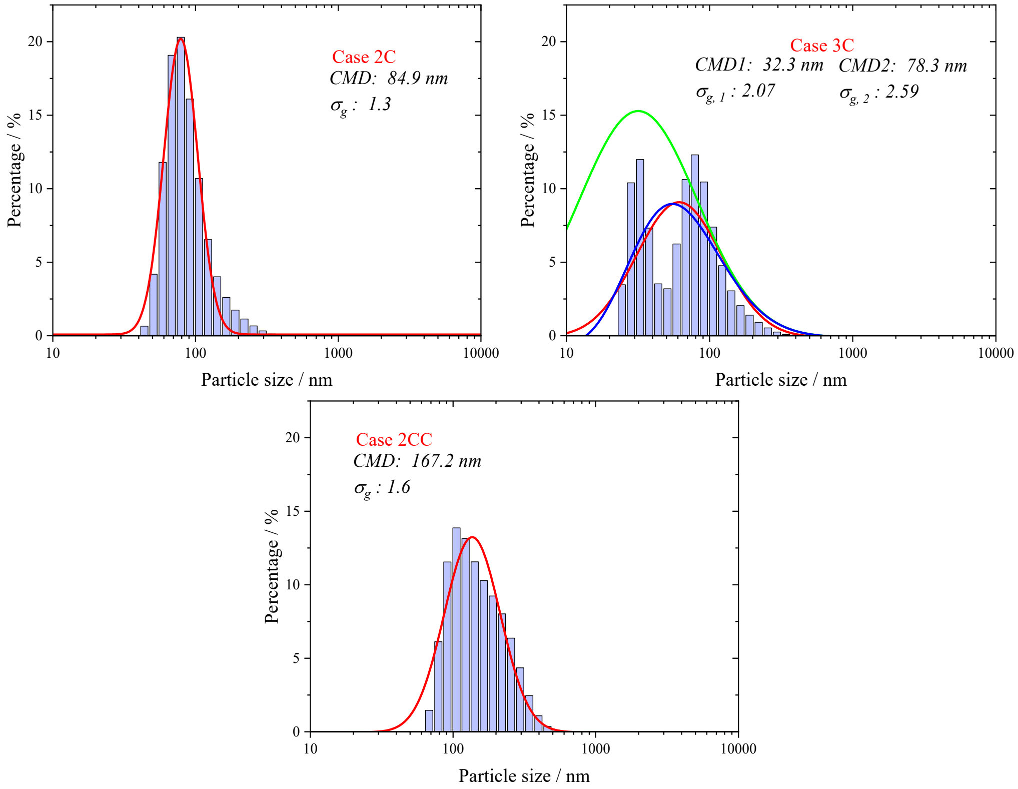


*Figure S3 (continuation). Particle size distribution from DLS. Lognormal deviations and median diameters shown for each case. Case 3C is fitted to a bimodal distribution.*

## S.4. Line profile from STEM images

Figure S4 shows the STEM image with the EDX-line employed to plot Figure S5. This STEM image corresponds to the sample of Case 3C.

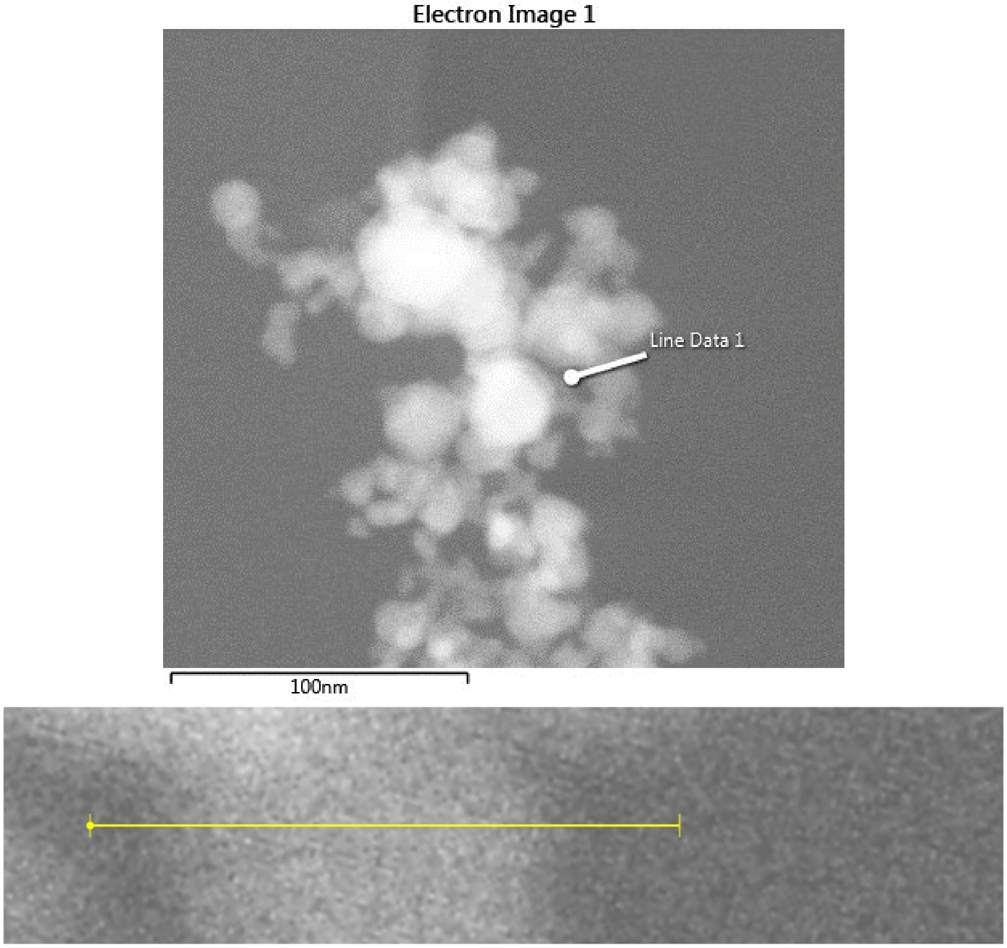


*Figure S4. STEM image of the coated nanoparticles from Case 3C plotted with line employed to create Figure S5.*

Figure S5 shows the elements (Si, O, Fe) for the line profile of Figure S4 (Case 3C). This figure confirms the presence of a core mostly made out of Fe and O ($Fe_xO_y$, shown to be magnetite/maghemite mainly by XRD) and a small part on the sides (coating) containing Si and O mostly ($SiO_2$). Similarly, Figure S7 and Figure S8 display the line profiles for Cases 1C, 2C, and 2CC.

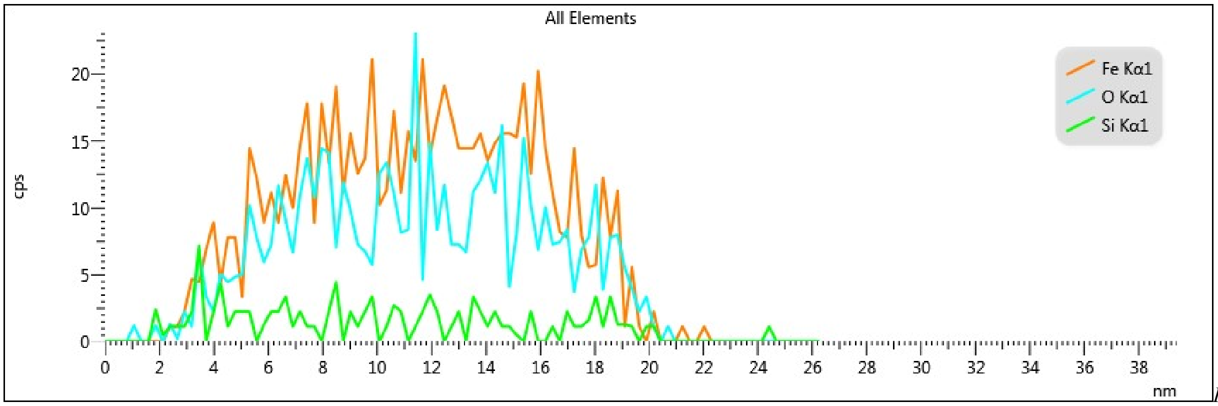


*Figure S5. Elements present on the line profile shown in Figure S4 for Case 3C.*

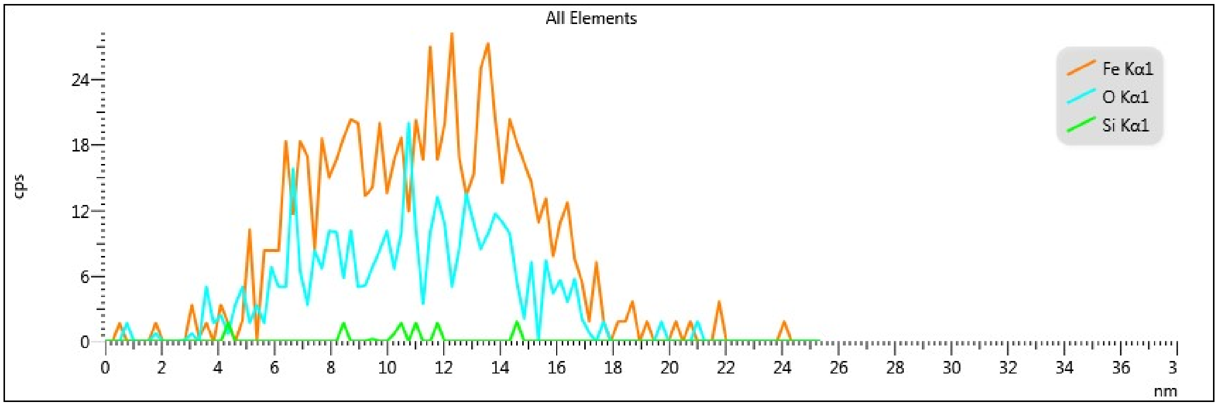


*Figure S6. Elements present on the line profile for Case 1C.*

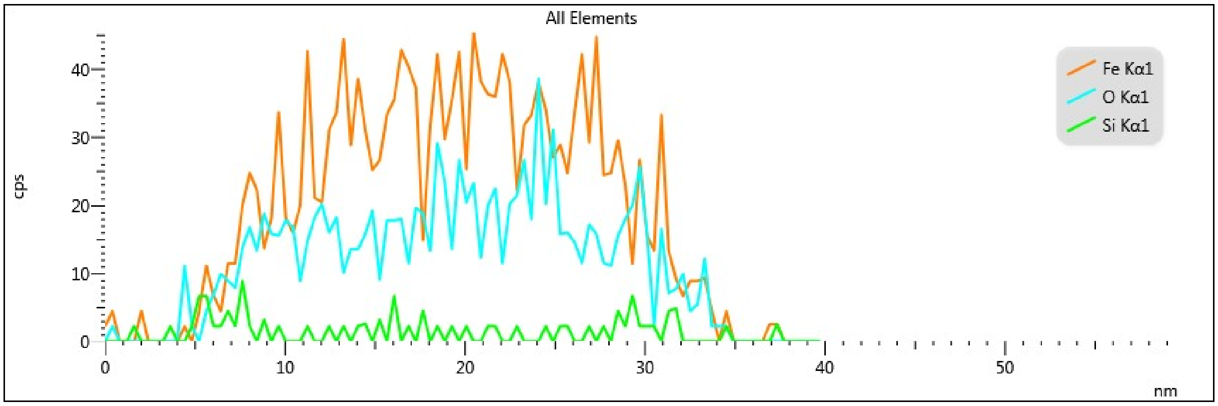


*Figure S7. Elements present on the line profile for Case 2C.*

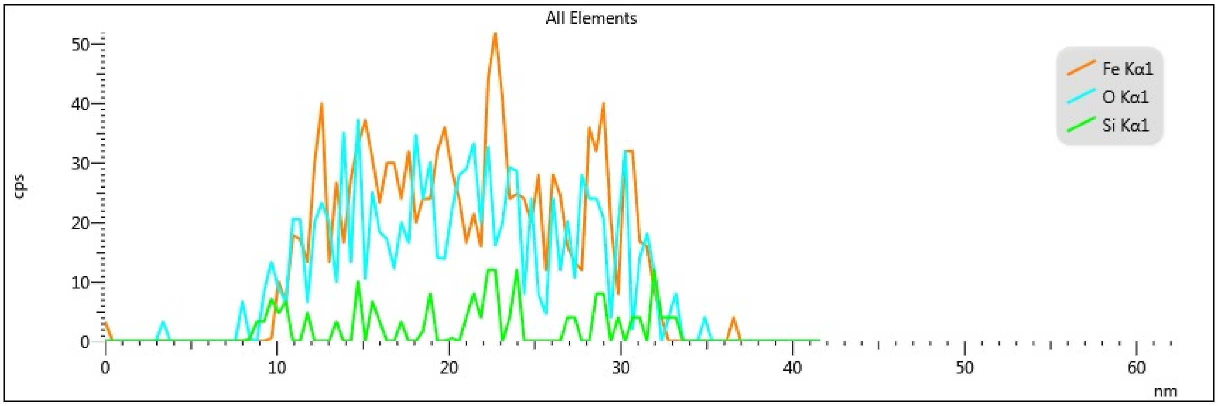


*Figure S8. Elements present on the line profile for Case 2CC.*

## S.5. XRD diffractograms

Figure S9 shows the XRD diffractograms and Figure S10 shows an example of the fitting for Case 2CC. Even though these diffractograms are practically the same for all cases, the fitting was done for maghemite and magnetite. Moreover, for the cases with coating, we also added the fitting for $SiO_2$ to see if it is possible to quantify the amorphous amount of silica as a coating agent (since homogeneous nucleation of silica in TEM was not detected for any of the coated cases). For the cases where $SiO_2$ was also fitted, a $SiO_2$ quartz sample was measured previously in the same device and used as a reference. For that measurement, we did a complete Rietveld refinement using the ICSD file for cristobalite (collection code 153886) according to the literature (DOI 10.1524/zkri.2011.1437). The obtained values for microstrain and crystallite size were 0.068 and 3.09 nm, respectively.

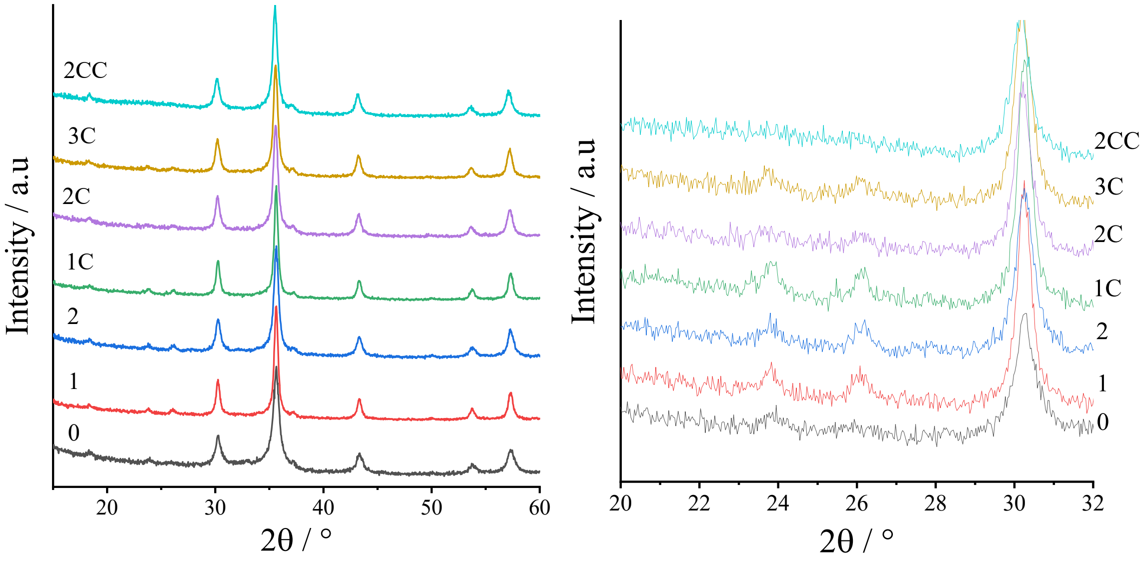


*Figure S9. XRD diffractograms obtained for both uncoated and coated cases (**left:** zoom-out, **right**: zoom-in).*

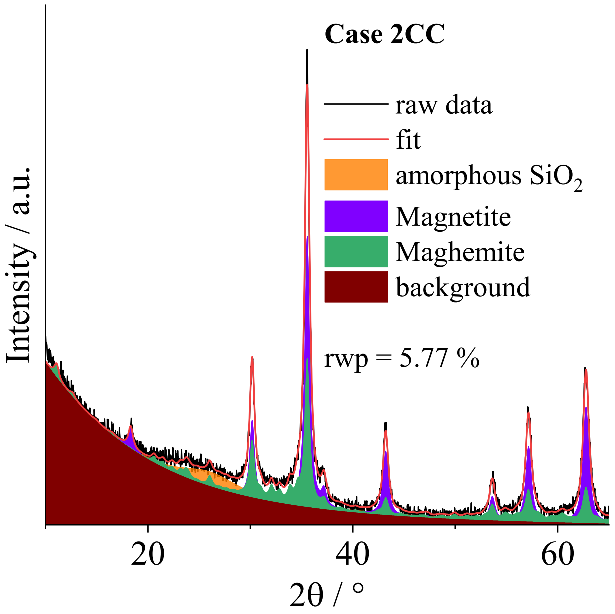


Figure S10. Example of XRD diffractogram fitting for Case 2CC.

The qualitative assessment of magnetite presence in the sample can only be done for Case 2CC, as it is present in a much larger quantity than in the other cases. For Case 2CC, the peaks at 23.8 and 26.2 degrees disappear, corresponding to maghemite phase. However, for the rest of cases, the mentioned peaks are still present given that the maghemite phase is much more predominant. A quantitative result for the magnetite and maghemite fractions in the samples based in XRD cannot be provided given (1) the similarities between both iron oxide phases and (2) the low presence of magnetite in all the samples.

For the "double-coated" Case 2CC, the amount of amorphous $SiO_2$ as calculated by this method is ≈19 wt %. This can be seen by a "hump" in the area of 20-30 degrees in Case 2CC (Figure S8). For the rest of cases with coating (Cases 1C, 2C, and 3C), the percentage for amorphous $SiO_2$ determined with this method is approx. 2 wt %, 6 wt %, and 7 wt %, respectively. For all uncoated samples, it was 0% as expected. Note that values below 10wt % are not reliable due to the detection limit of the method. Therefore, the only real difference in amorphous content is seen for Case 2CC, being the rest of Cases more or less similar to each other. Yet, it is interesting to observe that Cases 2C and 3C show slightly higher amorphous content and the highest is shown by Case 2CC, all in agreement with EDX. As shown in Table S1, the values for the $SiO_2$ in the samples — as calculated from XRD and the ones calculated from the SEM and TEM elemental analysis measurements — are close, with the differences being within the detection limit of the XRD.

Table S1. Silicon as measured from EDX (TEM and SEM), its corresponding silica, and the calculated silica as measured from XRD.

| | **Case** | **1C** | **2C** | **3C** | **2CC** |
|---|---|---|---|---|---|
| **EDX from TEM \| SEM** | **Si / at%** | 0.98 \| 3.6 | 6.2 \| 9.1 | 3.4 \| 7.9 | 12.6 \| 17.5 |
| **Calculation of corresponding $SiO_2$** | **$SiO_2$ / wt%** | 0.75 \| 2.8 | 4.8 \| 7.1 | 2.6 \| 6.1 | 9.9 \| 13.9 |
| **$SiO_2$ as calculated from XRD** | **$SiO_2$ / wt%** | 2 | 6 | 7 | 19 |

## S.6. Fourier-transform infrared spectroscopy (FTIR) spectra

The FTIR characterization was done for all coating cases. The FTIR spectra in Figure S11 show the expected characteristic signals for $SiO_2$ (Si-O asymmetric stretching bonding out-of-phase and in-phase at ~ 1200 $cm^{-1}$ and ~ 1050 $cm^{-1}$, and Si–OH bonds ~ 950 $cm^{-1}$), and the signals for mainly magnetite and maghemite ($Fe_3O_4$ and γ-$Fe_2O_3$, respectively), which are shown between 400 and 660 $cm^{-1}$ range. It was not clear if hematite (α-$Fe_2O_3$) signals at 540 and 470 $cm^{-1}$ were present, yet XRD showed that the samples did not contain hematite phase. The rest of possible iron phases signals occur between 1124 and 810 $cm^{-1}$ (based on Namduri et al. Corros. Sci. 50 (2008) and Nasrazadani et al. Corros. Sci. 34 (1993)), and they were not observed in any of the FTIR spectra, aligning with XRD results.

For Case 1C and 2C, the Si-related signals are very weak (extremely weak in Case 1C). That is attributed to the fact of having such a thin coating. Nonetheless, we could observe under the TEM that there was homogeneous coating of the IONPs for both cases (see Figure 3).

Figure S12 shows a comparison for the region where iron oxide signals are expected between the results from this work and the published literature from Cornell and Schwertmann "Iron Oxides in the Laboratory" (ISBN 3-527-30274-3). From Figure S12, it is already expected that Cases 2C, 3C and 2CC result in more magnetite than in Case 1C, which has been quantitatively confirmed by Mössbauer spectroscopy (see Table 1).

Apart from that, $SiO_2$ layers of alkoxysilanes are known to incorporate hydrocarbons to a significant extent. However, no major signals of stretching vibrations due to hydrocarbon incorporation are displayed between 3300 – 2850 $cm^{-1}$. This agrees with the elemental analysis of carbon from these samples, which shows that they contain a negligible amount of carbon (see Supplemental Information, Section S.7).

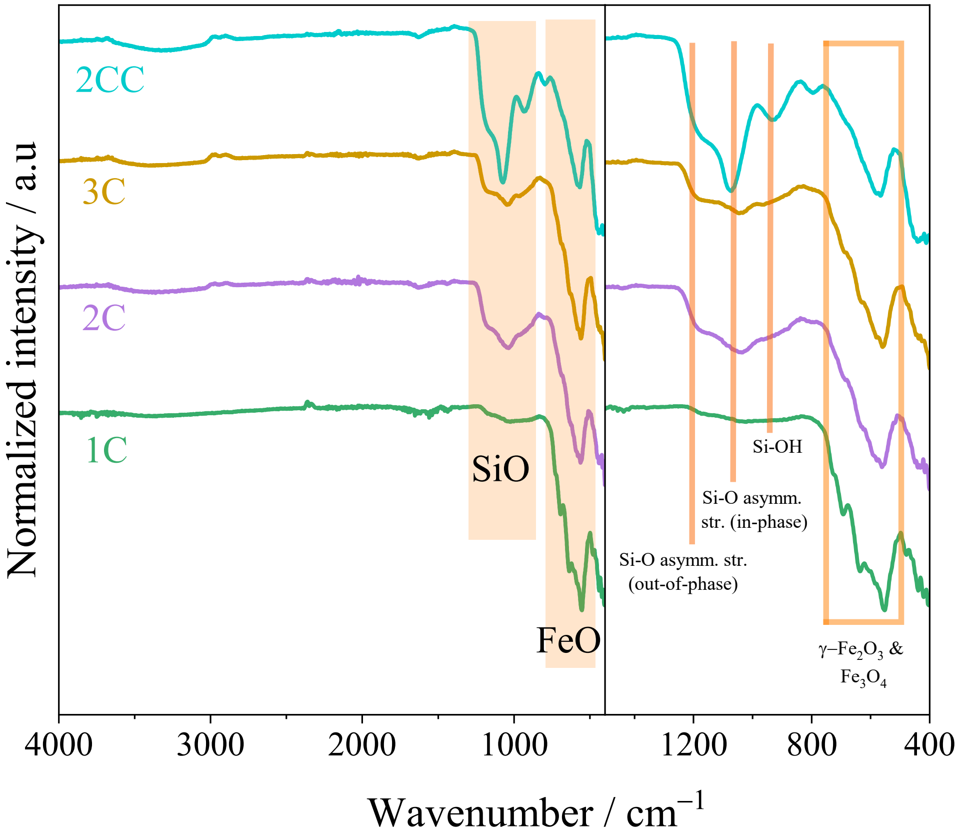


*Figure S11. FTIR for the coated IONPs nanoparticles for the wavelength region where Si- and Fe-related signals are expected (left: 4000 – 400 $cm^{-1}$; right: 1500 – 400 $cm^{-1}$). "str." abbreviation stands for stretching.*

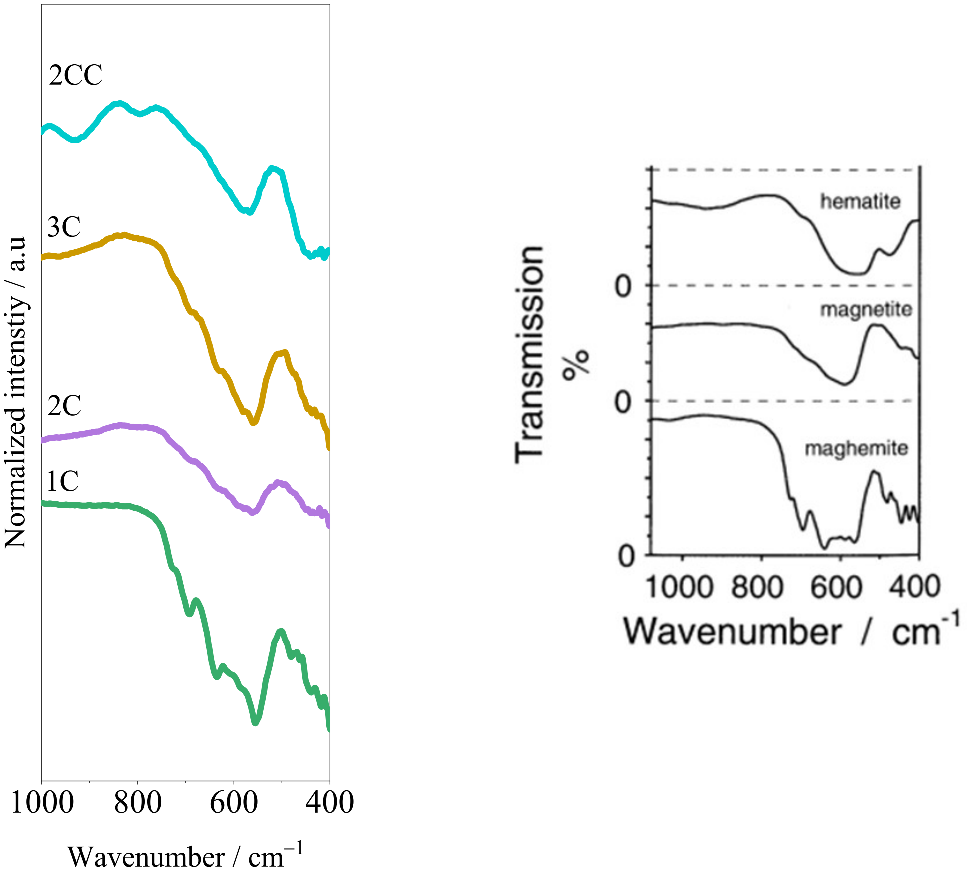


*Figure S12.**Left**: Zoom-in FTIR region from Figure S11 where the interest iron oxide signals are expected.*
***Right**: FTIR adapted from literature (Cornell and Schwertmann "Iron Oxides in the Laboratory" ISBN 3-527-30274-3).*

## S.7. Elemental content analysis

The carbon content analysis is carried out to check on the presence of hydrocarbon species in the nanomaterials produced (see Figure S11). The low amount of carbon (<1 % in weight) indicates that there is a negligible amount of carbonaceous species present in the synthesized nanomaterials in all cases.

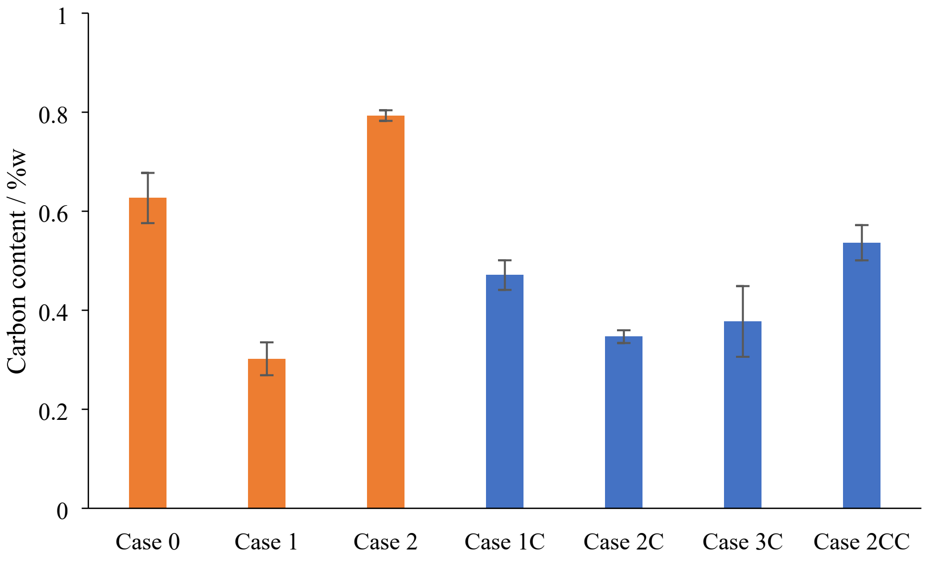


*Figure S13. Carbon content in each synthesized nanomaterial with quenching applied and without coating (orange) and with it (blue). See color references in the online copy.*

The oxygen (O), iron (Fe), and silicon (Si) elemental content has been measured by EDX/SEM and their results are shown in Table 1 and in a plot for in Figure S14. For Case 2CC, where the inlet of silica precursor was doubled (x2.5), there is an increase in atomic silicon (and consequently of oxygen) content as expected. This agrees with the thickness increase of the silica shell of the nanoparticles observed by TEM.

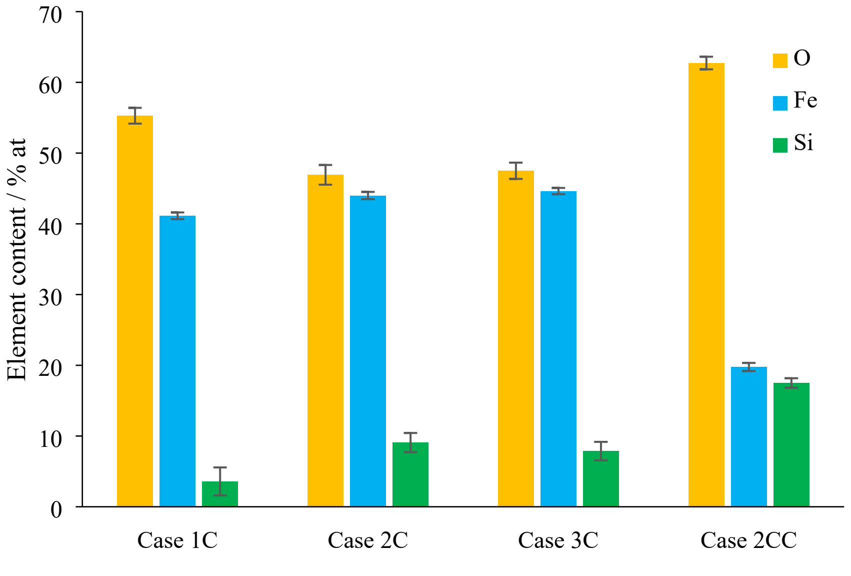


*Figure S14. Oxygen (yellow), iron (blue), silicon (green) content in each synthesized nanomaterial with coating applied. See color references in the online copy.*

## S.8. Magnetic characterization

Given that the nanoparticles are stored under normal conditions after being synthesized and magnetic properties of the synthesized IONPs are expected to change over time, Table S2 shows the time between the experimental synthesis and the magnetic characterization via Mössbauer spectroscopy (all of them being ≈ 2–3 weeks). Figure S15 displays the fitted Mössbauer spectra recorded at 5 K in a magnetic field of 8 T applied parallel to the γ-ray propagation direction.

Spin canting angles extracted from these in-field Mössbauer spectra are used to explain the peculiar trend in high-field magnetization observed in Figure S18. For uncoated particle powder samples, comprising of maghemite only within the error bar, we find spin canting angles of $\theta_{A0} = 22°$ and $\theta_{B0} = 32°$ for Case 0, $\theta_{A1} = 15°$ and $\theta_{B1} = 21°$ for Case 1, and $\theta_{A2} = 16°$ and $\theta_{B2} = 24°$ for Case 2. Estimating A- and B-site sublattice magnetization M relative to saturation $M_S$ via $M = M_S \cos(\theta)$ (see G.A. Petitt, D.W. Forester, Physical Review B, 4, 1971), we find a trend in magnetization values precisely reproducing the trend found experimentally for Cases 0, 1, and 2, as demonstrated in Figure S18, when assuming $\mu_{Fe3+} = 5\mu_B$ and considering the stoichiometry of maghemite $[Fe^{3+}]_A\left[\frac{5}{3}Fe^{3+}\frac{1}{3}\square\right]_B O_4$. It is evident that the stronger B-site spin canting, also often observed in literature for a majority of spinel systems, strongly reduces the magnetization of the dominant magnetic sublattice, whereby a higher degree of magnetic frustration as observed for case 0 results in a distinct reduction in net magnetization. This effect is likely connected to direct surface contact or sinter bridges due to high agglomeration/aggregation of the bare IONPS resulting from the specific setup configuration for Case 0 (i.e., no coating nor quenching applied). This highlights the relevance of having a quench gas injected right above the flame.

Following this approach, we were able to verify that the trend in magnetization of uncoated particles can be explained by different degrees of spin frustration, without the need to consider additional effects. Only when going to silica-capped particles do further mechanisms affect the high-field magnetization, primarily (i) enhanced magnetization by partial conservation of magnetite and (ii) reduced net magnetization by addition of silica material.

Table S2. Time period between each experimental synthesis and their magnetic characterization.

| Case | 0 | 1 | 2 | 1C | 2C | 3C | 2CC |
|---|---|---|---|---|---|---|---|
| **Configuration** | - | Option A | Options B/C | Option A | Option B | Option C | Option B |
| **Quenching nozzle** | OFF | ON | ON | ON | ON | ON | ON |
| **Coating nozzle** | OFF | OFF | OFF | ON | ON | ON | ON (x2) |
| **Dates** | | | | | | | |
| **Time passed between synthesis and Mössbauer spectroscopy (days)** | 9 | 26 | 34 | 16 | 14 | 20 | 11 |

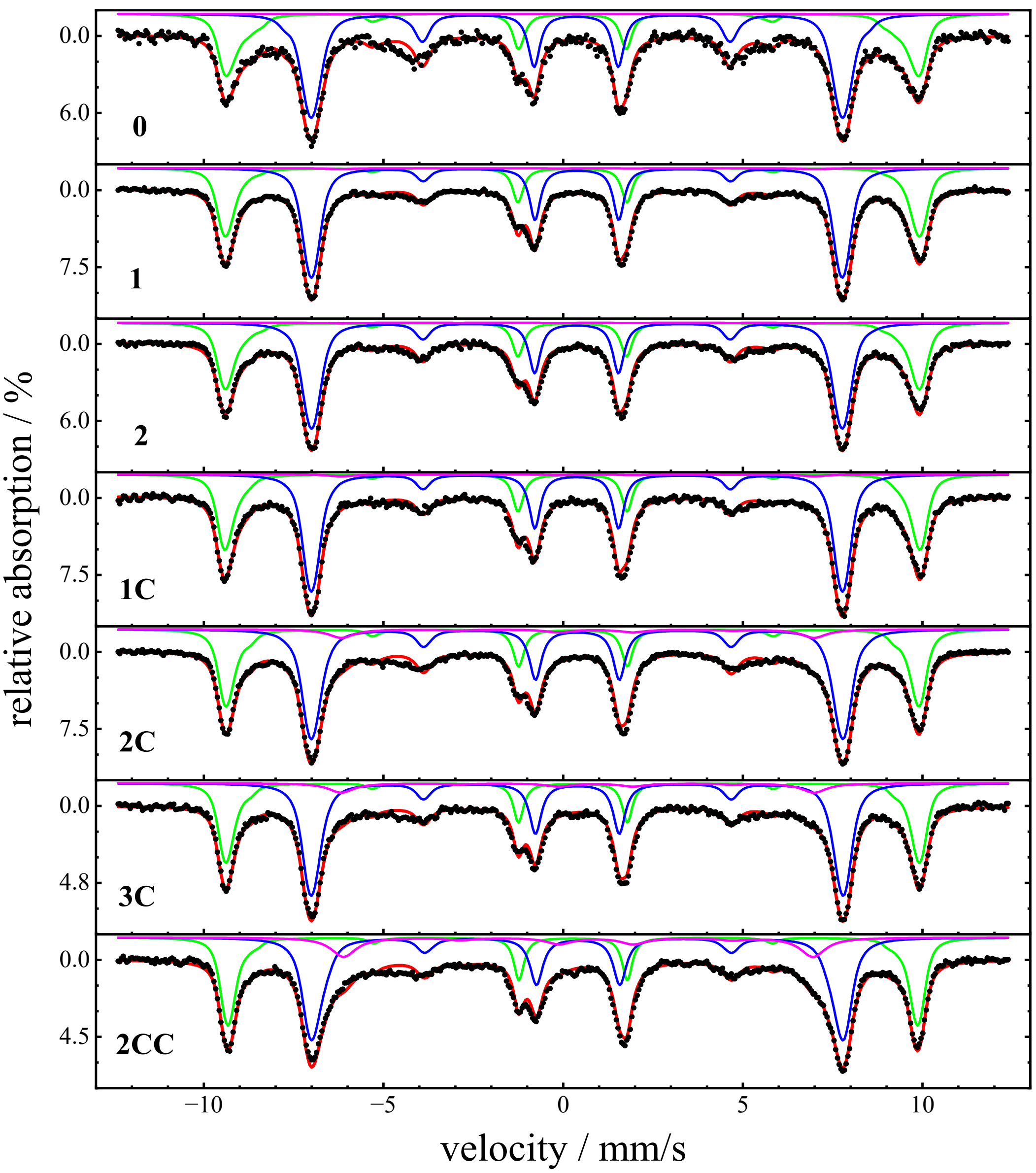


*Figure S15. Mössbauer spectra recorded at 5 K in a magnetic field of 8 T applied parallel to γ-ray propagation direction (red/dots), showing contributions of A-site (green) and B-site $Fe^{3+}$ (blue), and B-site $Fe^{2+}$ (pink). See color references in the online copy.*

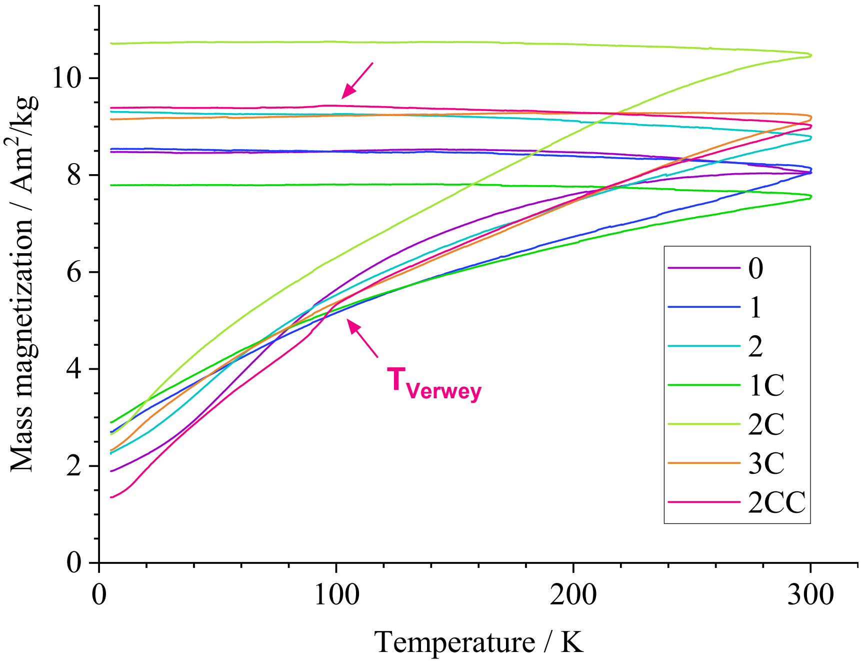


*Figure S16. Zero-Field-Cooled/Field-Cooled (ZFC-FC) magnetization measurements recorded at 10 mT. Arrows mark the position of the Verwey temperature in sample 2CC. See color references in the online copy.*

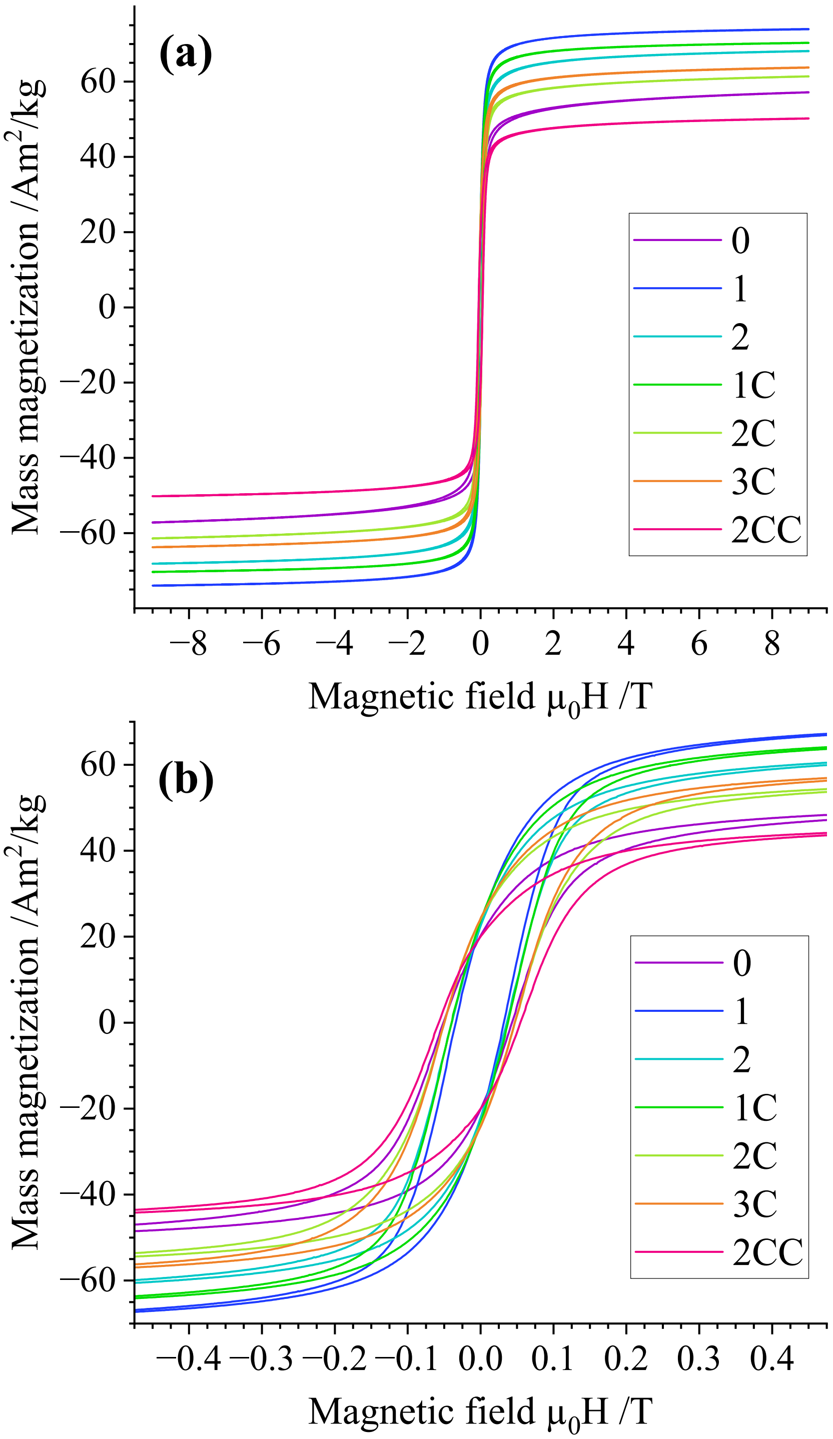


*Figure S17. Field-dependent magnetization curves M(H) recorded at 5 K (a), shown in (b) zoom-in. See color references in the online copy.*

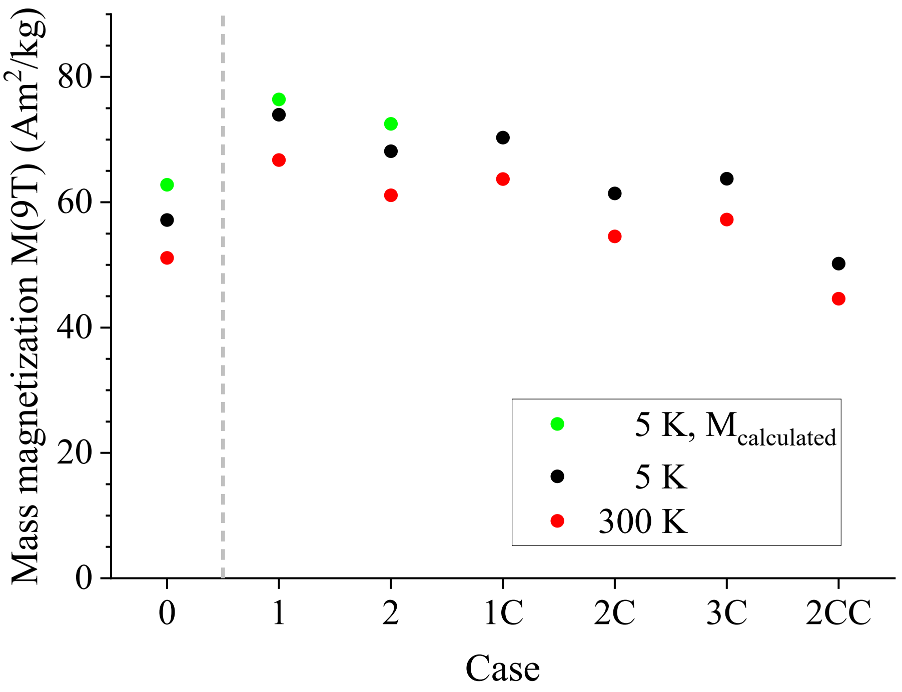


*Figure S18. Experimentally determined (black, red) and calculated (green) high-field magnetization ($M_{calculated}$) at 9 T, with the latter obtained as described in the text, using spin canting angles extracted from Mössbauer spectroscopy. The dashed line separates the case without coating or quenching (Case 0) from the rest of cases with only quenching (Cases 1 and 2), and with coating and quenching (Cases 1C, 2C, 3C, and 3CC).*

## S.9. SEM/EDS characterization

The characterization with a Scanning Electron Microscopy/Energy Dispersive Spectrometer (SEM/EDS) of the surface of the samples should provide further understanding on the morphology and elements present. The EDS quantification of oxygen (O), silicon (Si), and iron (Fe) were obtained from an sample area about 250 µm x 300 µm. The results for the elemental presence of O, Si, and Fe (in at.%) are reported in Table 2, and are similar to the ones obtained from TEM/EDX characterization. The images of the surface morphology for all cases can be seen in Figure S19.

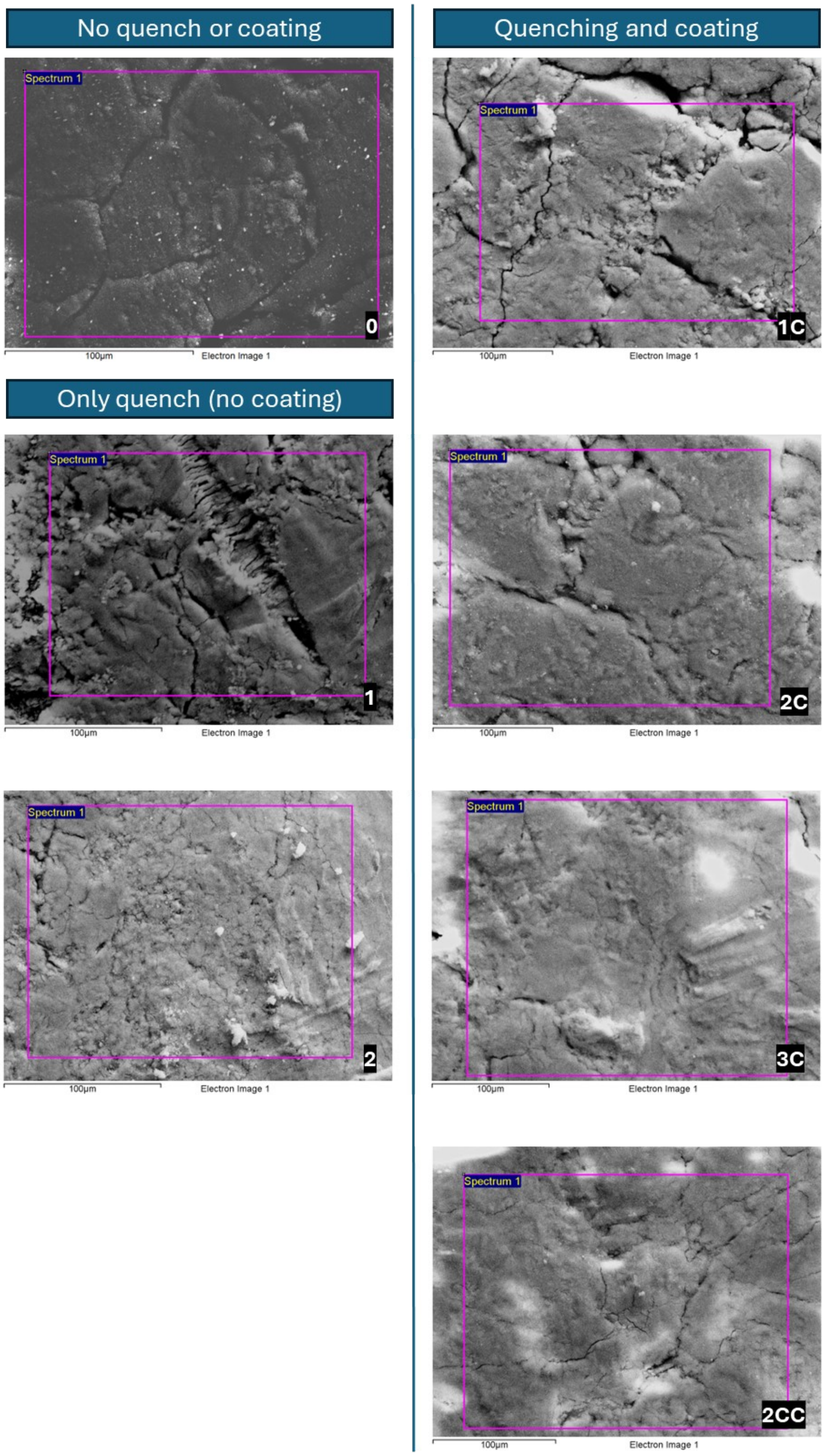


*Figure S19. SEM images of the powder's surfaces for all study cases. Number of study case shown in the bottom-right corner of each image.*
